# Crossing Barriers: Advancements in Macromolecular Therapeutics for Neurodegenerative Diseases and Strategies to Overcome the Blood–Brain Barrier

**DOI:** 10.1021/acsptsci.5c00153

**Published:** 2025-07-14

**Authors:** Bekas Alo, Christina Lamers

**Affiliations:** Institute for Drug Discovery, Faculty of Medicine, 70622 Leipzig University, Leipzig 04103, Germany

**Keywords:** blood−brain barrier, neurodegenerative disease, Alzheimer’s disease, antibody therapeutics, central nervous system, peptide therapeutics

## Abstract

Neurodegenerative diseases, such as Alzheimer’s disease, Parkinson’s disease, and amyotrophic lateral sclerosis, present considerable challenges for our societies and health systems due to their progressive nature, the demographic shift toward older populations, and limited treatment options. Recent advances in macromolecular therapeutics, including antibodies, peptides, and proteins, offer novel therapeutic modalities for a broad range of diseases. Their high potency and specificity hold promise for disease-modifying therapies to combat neurodegenerative diseases. However, the blood–brain barrier poses a significant challenge for the effective delivery of these large molecules to the central nervous system. This review discusses the physiological role of the blood–brain barrier and its influence on restricting the exposure of macromolecules in the brain. Furthermore, emerging strategies for enhancing blood–brain barrier permeability to macromolecules are highlighted. This review summarizes modifications designed to utilize receptor-mediated uptake, adsorptive-mediated transcytosis, carrier-mediated transport, and nanoparticle-based delivery systems to overcome the blood–brain barrier. Additionally, we emphasize the importance of testing macromolecular therapeutics for their blood-brain barrier permeability and review the methods for such *in vitro* and *in vivo* testing. Finally, we shed light on therapeutics in preclinical and clinical development for neurodegenerative diseases and their challenges.

## Graphical abstract

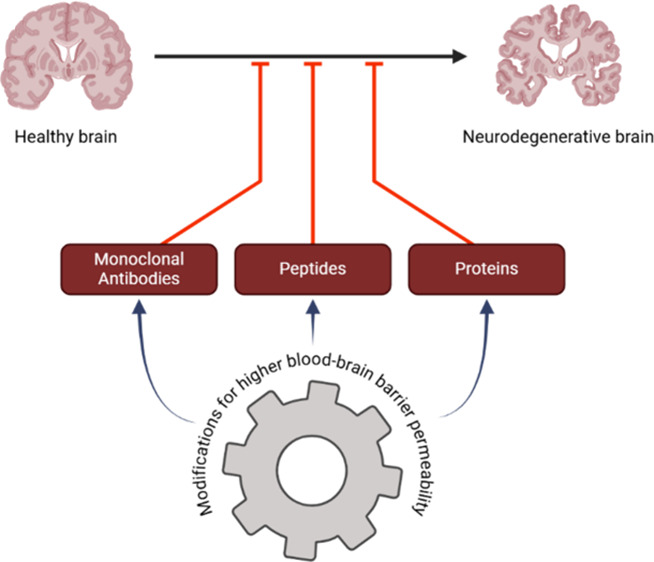

Neurodegenerative diseases, encompassing conditions such as Alzheimer’s disease (AD), Parkinson’s disease (PD), and amyotrophic lateral sclerosis (ALS), pose an enormous challenge to public health worldwide, now and in the years to come.^1–3^ These disorders are characterized by progressive degeneration of the nervous system's structure and function, leading to debilitating cognitive and motor impairments.^4–7^ Current treatments for neurodegenerative diseases are symptom-relieving small molecules, such as acetylcholinesterase inhibitors (e.g., donepezil), NMDA receptor agonists (memantine), or dopamine receptor agonists (e.g., ropinirole); however, they lack disease-modifying activities. In recent years, there has been growing interest in disease-modifying macromolecular therapeutics, including antibodies, peptides, and proteins, as promising avenues for treatment. Antibodies, with their high specificity and affinity, hold promise for targeted interventions against neurodegenerative diseases.^8–10^ Aducanumab (discontinued), lecanemab, and donanemab are monoclonal antibodies (mAbs) approved by the Food and Drug Administration (FDA) for AD treatment. Further macromolecular modalities in development include proteins^11–13^ and the so-called new modalities (oligonucleotides, macrocycles, and peptides).

However, the translation of macromolecular compounds with *in vitro* activity into viable therapies is hindered by poor blood–brain barrier (BBB) permeability. The BBB acts as a gatekeeper, tightly regulating the passage of molecules between the bloodstream and the brain parenchyma.^14^ While this barrier is crucial for maintaining the delicate homeostasis of the central nervous system (CNS), it presents a major obstacle for the delivery of therapeutic agents into the brain. While there are rules of thumb for small molecules to achieve passive BBB permeation, macromolecules, in particular, show a limited ability to traverse the BBB passively.^15–18^ Several strategies for improving BBB permeability of macromolecules, including the use of receptor-mediated transcytosis (RMT), adsorptive-mediated transcytosis (AMT), carrier-dependent transport (CMT), and nanoparticle (NP)-based delivery systems, have been successfully utilized in preclinical and clinical studies. This article provides an overview of the physiology of the BBB and the mechanisms of transport of molecules across the BBB. Macromolecular therapeutics currently in development for the treatment of neurodegenerative diseases are discussed, with a focus on their BBB permeability. Furthermore, the challenges of testing BBB permeability are discussed, and emerging strategies for enhancing the delivery of macromolecules to the CNS by chemical modifications are highlighted.

## 1 The CNS Is Protected by the BBB

The BBB covers nearly all capillary vessels in the brain, shielding the brain parenchyma from the bloodstream and its contained compounds and stringently regulating transport from and into the brain. It consists of three types of cells: brain endothelial cells (BECs), pericytes (PCs), and astrocytes (ACs). BECs represent the first barrier from the luminal site of the capillary vessels.^19^ They are rich in tight junctions and other cellular junction molecules, resulting in nonexistent passive paracellular transport of soluble compounds from the blood to the brain and vice versa, in contrast to capillaries in the periphery.^20^ Low expression levels of proteins needed for vesicular transport and fluid-phase transcytosis,^21^ for example, the vesicle-associated protein caveolin-1,^22^ a dense, highly negatively charged glycocalyx,^23^ and high expression levels of efflux transporters, such as P-glycoprotein (P-gp, also known as multidrug resistance protein 1, MDR1) reduce transcellular transport. Transport of essential brain nutrients to the brain and brain metabolites from the brain is specifically enabled by specific transporters on BEC membranes^20,24^ (Table 1).

**Table 1:** Endogenous BBB Transporter

transport mechanism	transporter	substrates
carrier-mediated transport (CMT)	GLUT1	glucose
	LAT1	large neutral amino acids (e.g., leucine)
	CAT1	cationic amino acids (e.g., arginine)
	MCT1	short-chain monocarboxylates (e.g., L-lactate)
	CNT2	purine nucleosides and uridine
	CHT	choline
active efflux transport	ABC transporter family (ABCA1, ABCB1 (also known as P-gp or MDR1), ABCC4 and ABCG2)	various substances with different properties (e.g., cholesterol, vincristine, cyclosporine A, or topotecan)
	OAT3	organic anions (e.g., *para*-aminohippurate or indoxyl sulfate)
	EAAT-1, -2, -3	anionic amino acids (e.g., aspartate)
	TAUT	taurine
receptor-mediated transport (RMT)	insulin receptor	insulin
	transferrin receptor (TfR)	transferrin
	insulin-like growth factor receptor (IGF_1/2_R)	insulin-like growth factor and insulin
	leptin receptor (LepR)	leptin
	neonatale Fcγ-receptor (FcRn)	Fc fragment of IgGs
	low-density lipoprotein receptor (LDL receptor)	low-density lipoprotein
	LDL receptor-related protein 1 (LRP-1)	ApoE, lipid-related ligands, protease, protease inhibitor complexes and different proteins (e.g., α2-macroglobulin, β-amyloid1–40, apamin, soluble melanotransferrin, lactoferrin)

PCs share a basement membrane with BECs on the abluminal site,^25,26^ contributing to the BBB, and they also have direct contact with the endothelial cells through peg-and-socket junctions.^27,28^ PCs are recognized for their role in the formation of the BBB during embryogenesis, e.g., by secreting platelet-derived growth factor BB.^29^ Additionally, they control the expression of efflux transporters and junctional proteins of BECs, thereby regulating BBB permeability and transporter function during adulthood.^27,30^

ACs are a subset of glial cells that cover the capillary vessel wall from the abluminal site.^31^ Through their interactions with PCs, they regulate blood flow in response to neuronal activity.^32^ While ACs may not directly participate in the development of the BBB,^29^ laminin α2, glial-derived neurotrophic factor, and Sonic hedgehog from ACs have been shown to sustain the proper function of the BBB by increasing junctional protein expression in BECs and, thus, reducing BBB permeability.^33^ Additionally, motile microglia, regulating capillary permeability and cerebral blood flow,^25,34^ also influence the BBB.

Most areas of the brain are shielded by the BBB, while the so-called circumventricular organs (consisting of the pineal gland, hypothalamus, subfornical organ, subcommissural organ, area postrema, posterior pituitary, and vascular organ of the lamina terminalis) have fenestrated vasculature^35^ lacking the BBB. This enables the exchange of molecules, such as endogenous peptide hormones, between the brain, blood, and cerebrospinal fluid (CSF), which is essential for sensory and homeostatic functions^36^ or cardiovascular regulation.^37^ Meanwhile, macromolecules might reach the CSF via passage through the circumventricular organs, which does not necessarily imply substantial exposure in the brain parenchyma, as exemplified by blood-borne macromolecules, such as IgG or albumin. These are detectable in the CSF, but they still encounter the challenge of crossing the CSF–brain interface. The CSF–brain interface^38–40^ and the CSF–spinal cord parenchyma interface^41^ are regulated by ependymal cells, which are less restrictive than BECs, but still pose a barrier.

BBB dysfunction due to dysfunctional expression of tight junctions and transport proteins, endothelial degeneration, and pericyte degeneration has been observed in several patients with neurodegenerative diseases. However, it remains unclear whether the dysfunction is a cause or a consequence of the neurodegeneration. Given the abundance of comprehensive reviews and research articles on the function and physiology of the BBB and CSF in both healthy and diseased states,^14,42–46^ we will not delve into this matter in depth.

## 2 Routes across the BBB and Their Role in Transporting Macromolecular Therapeutics

One important consideration during the development of therapeutic compounds for CNS diseases is the optimization of CNS exposure to the therapeutic. This is directly correlated to the molecular modality of the therapeutic, as the physicochemical parameters of the molecule determine brain parenchyma exposure. The first therapeutic compounds targeting the CNS were small-molecule therapeutics for the treatment of depression and neuroleptic diseases. These CNS-active small molecules have in common a high lipophilicity and a secondary or tertiary amine, leading to a high fraction of uncharged molecules at physiological pH, enabling passive diffusion. Based on observations by Lipinski, small molecules can cross membranes passively due to their low molecular weights (<500 Da), reduced number of hydrogen bond donors and acceptors, and lipophilicity (<log*P* 5). In addition, exposure of the brain to compounds is influenced by their susceptibility to efflux pumps, such as P-gp. Peptides, nucleic acid modalities, proteins, and antibodies can be described as molecular modalities with high molecular weights, a high number of hydrogen bond donors and acceptors, charged groups, and therefore overall hydrophilicity; thus, they face bigger hurdles to achieve brain exposure.

### 2.1 Passive Diffusion

One of the most common and extensively studied mechanisms used by therapeutic molecules to cross the BBB is passive diffusion.^47^ This is an energy-independent and nonsaturable transport through the brain’s microvascular endothelial barrier and adjacent cells following the concentration gradient of the molecules. Based on the wealth of data acquired for CNS-active small molecules, several molecular characteristics, including lipophilicity, number of hydrogen bonds, total charge, molecular weight (MW), and molecular volume, have been shown to play critical roles in a molecule’s ability to traverse the BBB through the passive diffusion mechanism.^48–51^ These parameters and their cutoffs are derived for small molecules and, therefore, are not necessarily suitable for predicting membrane permeability to macromolecules. However, certain rules of thumb hold true. Passive transcellular diffusion is a method for rapid delivery into the brain. The rapid effects of ethanol and caffeine are attributable to this mechanism.^52^ For effective passive diffusion, the molecules need to interact with the lipid bilayer of endothelial cells, which is achieved if the compound exhibits lipophilicity values (LogP or LogD) of 0–3.5.^53^ However, it is important to note that increasing lipophilicity also increases the risk of being extruded by the BBB’s efflux pump system^54^ (Table 1), besides its detrimental effect on water solubility. Membranes are passively permeable to compounds with a maximum MW of approximately 450 Da, which corresponds to roughly 90 A^2^ molecular volume.^55^ MW and lipophilicity influence passive BBB permeability, leading to reduced passage of molecules with high MW, such as epipodophyllotoxin and vincristine, although they show a LogP value of 2.5.^56,57^

Due to the slightly acidic brain parenchyma,^49^ moderately basic compounds are preferred for uptake into the brain parenchyma over slightly acidic compounds,^58^ which might get trapped in the lipophilic membrane due to the equilibrium shifting toward the noncharged form.

The hydrogen bonding properties of a molecule also influence whether it can cross the lipid membrane and partition into the brain’s interstitial fluid. A neuronal network trained on CNS-active and CNS-inactive small molecules identified descriptors for predicting the CNS activity. Increasing the number of hydrogen bond acceptor groups (with a maximum of 30) or reducing the number of hydrogen bond donor groups (with a maximum of 20) decreased the ability of small molecules to be CNS-active.^59^ While this observation may apply to molecules within the MW range of 60–600 Da, hydrogen bonding becomes a particularly relevant factor for macromolecules, such as peptides and proteins.^60^ Their polymeric structure, consisting of an amide backbone that serves as both a hydrogen bond donor and acceptor, makes them unlikely to passively cross the BBB. Furthermore, antibodies, proteins, and peptides have MWs that span from low kDa (peptides) to as high as 150 kDa for mAbs and are therefore beyond the typical range of passively permeable compounds.

Nevertheless, there are endogenous peptides and small proteins that have been documented to cross the BBB through passive diffusion, including the delta-sleep-inducing peptide,^61^ α-MSH,^62^ orexin A,^63^ nesfatin-1,^64^ and neuropeptide Y.^65^ They form a secondary structure that renders them more lipophilic due to intramolecular hydrogen bonds, as exemplified by the β-turn-rich delta-sleep-inducing peptide^66^ and the hairpin-structured α-MSH.^67^ Consequently, the number of hydrogen bonds does not generally limit the membrane permeability of macromolecules.

### 2.2 Extracellular Pathways

The brain parenchyma is accessible to macromolecules through the fenestrated blood vessels of the circumventricular organs. This is generally referred to as the “extracellular pathway”^68^ (Figure 1). This pathway provides access to the subarachnoid space, pial surface, and circumventricular organs, enabling these macromolecules to enter the brain after surpassing the CSF-brain interface.^69^ The rate of uptake into the brain through extracellular pathways is notably slow, with only a modest quantity of administered macromolecules reaching the brain parenchyma via this route.^68^ Consequently, only macromolecules with extended half-life and high potency will accumulate in the brain at a concentration sufficient to elicit *in vivo* activity. Liraglutide, a glucagon-like peptide-1 receptor agonist used in the treatment of type 2 diabetes,^70^ is known to mediate weight loss effects by acting in the CNS.^71,72^ The extended half-life and high potency of liraglutide allow this peptide to be transported into the brain via the extracellular pathway.^73^ Furthermore, antibodies with a long circulation half-life, e.g., due to the neonatal Fc receptor-mediated cycling,^74^ can accumulate in the brain via the extracellular pathway. This holds especially true for therapeutic antibodies, which are at a much higher serum concentration compared with endogenous IgGs, leading to substantial exposure in the CSF and, partly, in the brain parenchyma, while endogenous antibodies are usually not found in the CSF or brain. In the case of mAb, typically 0.1% of an i.v.-injected dose reaches the brain, and this number serves as a typical benchmark^75–79^ for mAb exposure in the brain; however, much lower concentrations have also been reported (0.009% detected in the cortex and 0.0017% detected in the hippocampus).^80^ Immunostaining studies conducted by Broadwell and Sofroniew^69^ demonstrated that in rats, intravenously injected mouse IgGs were present on the pial surface. However, whether the extracellular pathway is the only pathway for therapeutically applied IgGs to enter the brain is still under debate. IgGs have also been observed to enter the brain through spontaneous endocytosis and RMT, which is explained later.

**Figure 1:**
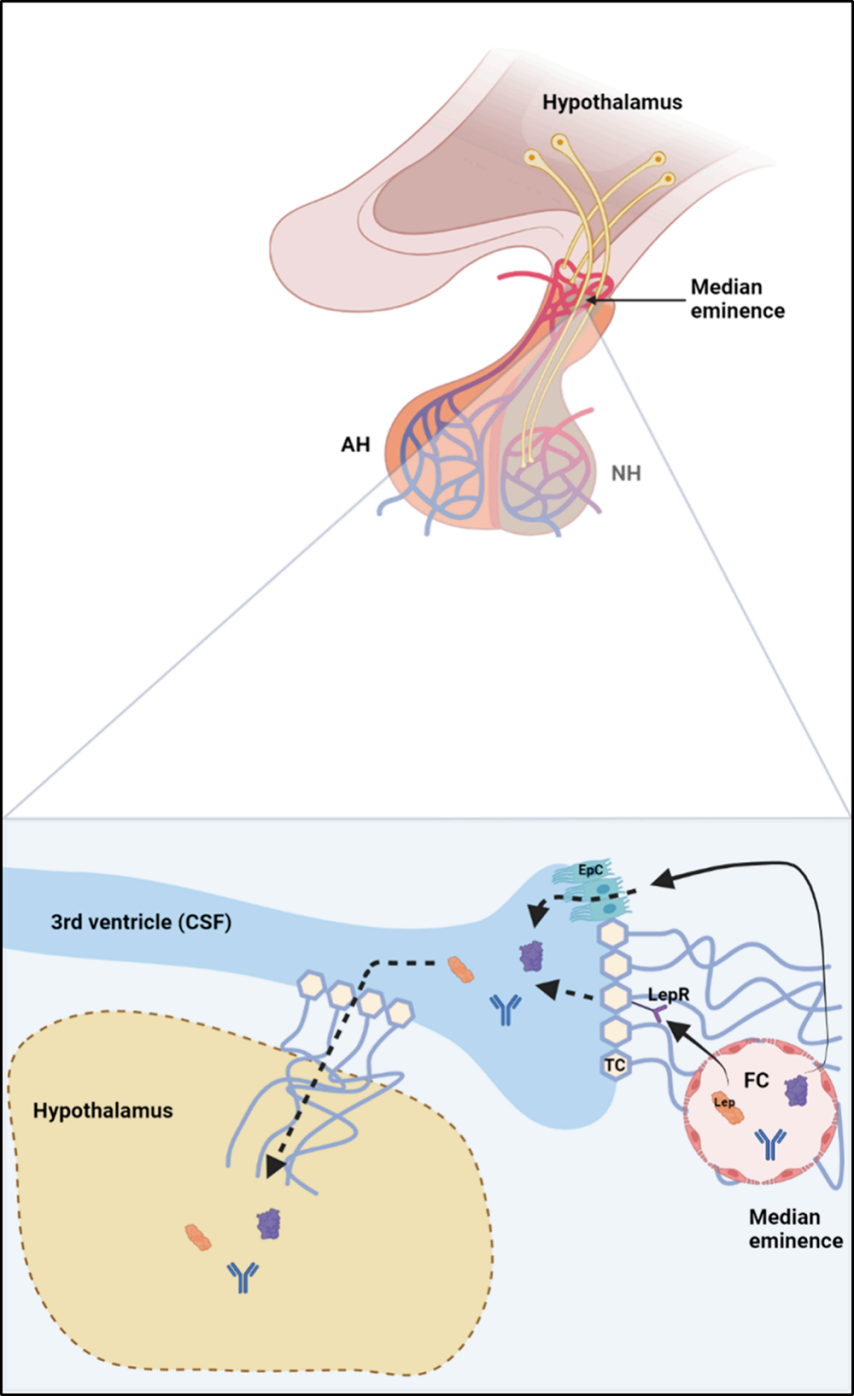
Extracellular pathway through fenestrated endothelial cells of the median eminence as a representative member of the circumventricular organs. AH, adenohypophysis; NH, neurohypophysis; FC, fenestrated capillaries; Lep, leptin; LepR, leptin receptor; EpC, ependymal cell; TC, tanycytes; and CSF, cerebrospinal fluid. Created in BioRender.

More recent studies have demonstrated that the endogenous peptide leptin can traverse the blood vessels of the median eminence via the extracellular pathway and be absorbed into the CSF, although they are mediated by tanycytes (TC) in a leptin-receptor-dependent manner.^81^ TCs are specialized epithelial cells that surround various circumventricular organs, including those separating the median eminence and regions of the hypothalamus from the CSF of the third ventricle. They are also likely responsible for leptin reaching the hypothalamus^81,82^ (Figure 1).

Even though only a small fraction of circulating macromolecules may reach the CNS or the CSF via the extracellular pathway,^68^ prolonged half-life, low distribution volumes, and high potency enhance the exposure of macromolecules to the CNS using the extracellular pathway.

### 2.3 Adsorptive-Mediated Transcytosis

AMT is a vesicle-mediated mechanism that transports strongly positively charged molecules into the brain (Figure 2). This is achieved by their nonspecific interaction with the negatively charged glycocalyx on clathrin-coated pits,^83,84^ of which BECs exhibit a high density on the luminal cell membrane.^85^ It is recognized that AMT can become saturated, as the proteoglycans can act as “receptors” for a broad range of positively charged macromolecules.^86^ However, saturation is observed at very high concentrations due to its thousand-fold higher binding capacity compared with receptor-mediated processes.^84^ Additionally, a vast number of positively charged molecules can lead to an even further increase in uptake.^87^ One hypothesis is that a high number of vesicles merge to create channels across the BECs, leading to enhanced uptake of a higher amount of the AMT-causing substance.^49,88^ This phenomenon is similar to the observed mechanism of membrane-disrupting, mostly amphipathic antimicrobial peptides,^89–91^ which show high structural similarity to many AMT-causing molecules. Several studies have demonstrated that amphipathic antimicrobial peptides with a positive charge density exceeding a threshold of 31% are more likely to stabilize transmembrane pore or channel formation.^92,93^ Many pathogens (e.g., human immunodeficiency virus 1 (HIV-1)^94^ and the rabies virus^95^) have been reported to use highly positively charged proteins to reach the CNS using AMT.

**Figure 2:**
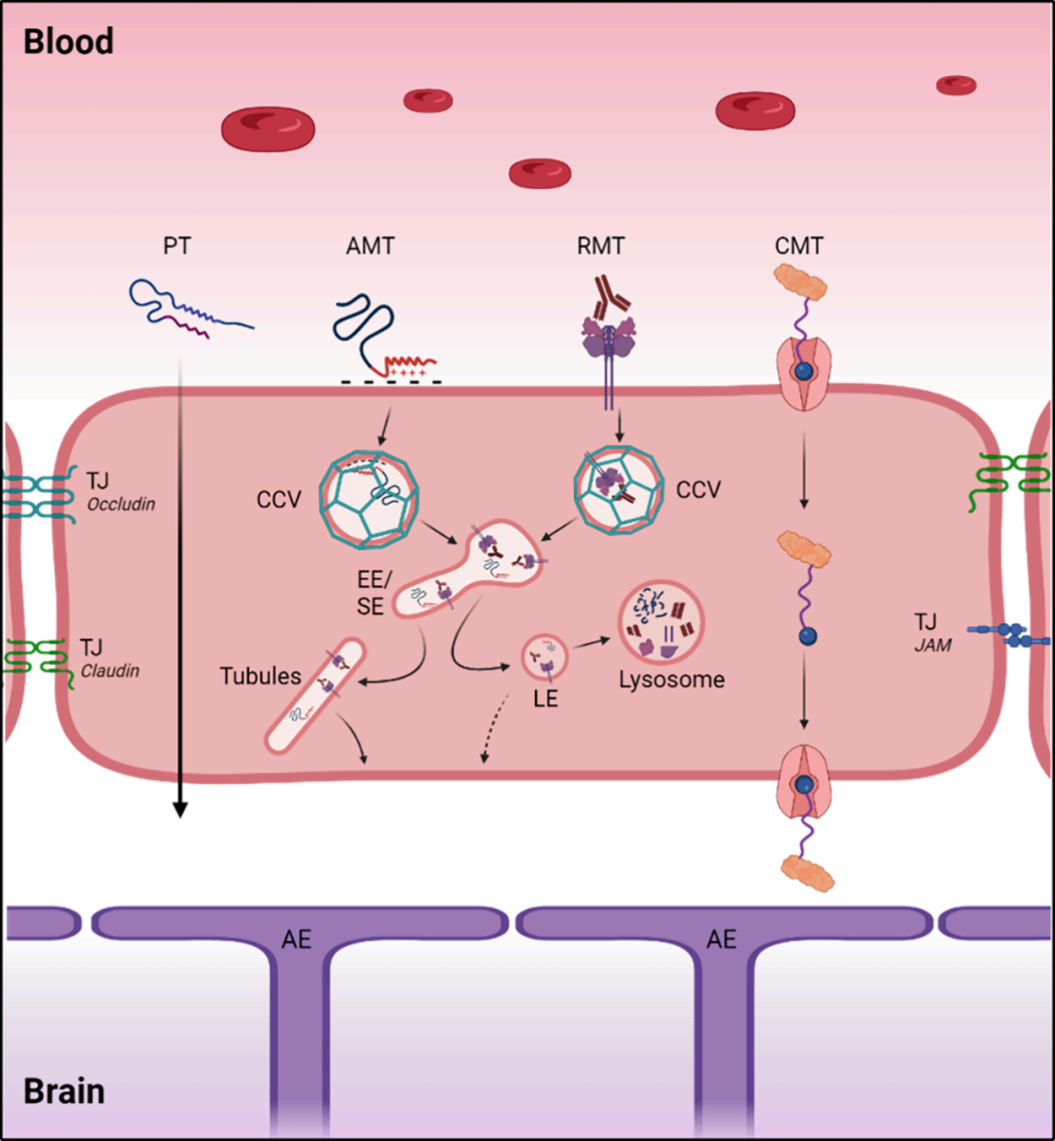
Mechanism of transport through BECs. PT, passive transport; AMT, adsorptive-mediated transcytosis; RMT, receptor-mediated transcytosis; CMT, carrier-mediated transport; TJ, tight junction; CCV, clathrin-coated vesicle; EE, early endosome; SE, sorting endosome; LE, late endosome; AE, astrocyte end feet. Created in BioRender.

### 2.4 Receptor-Mediated Transcytosis

RMT, like AMT, is a clathrin-dependent mechanism that is physiologically utilized to transport blood-borne macromolecules into the brain via binding to their membrane receptors on the luminal surface.^96,97^ Common examples are insulin binding to the human insulin receptor,^98^ low-density lipoproteins (LDLs) binding to LDL receptor-related protein 1 and 2 (LRP-1 and -2),^99^ leptin binding to the leptin receptor,^100^ and transferrin binding to the transferrin receptor (TfR)^101^ (Table 1). Furthermore, RMT has been reported as one possible mechanism for immune globulins to enter the brain in addition to the extracellular pathway (see above). St-Amour et al.^80^ demonstrated saturable uptake of human polyclonal Igs into the brain when injected peripherally into mice, competing with IgG for uptake. Potential receptors for Igs are the neonatal Fc receptor^102^ and LRP-1.^103^ However, other *in vitro* studies suggest the brain uptake of IgGs through spontaneous fluid-phase endocytosis, independent of any receptors.^104^

In the molecular mechanism of RMT, the ligand–receptor complex is internalized into cells via clathrin-coated pits (Figure 2). Once the ligand–receptor complex has been endocytosed, the vesicle (endosome) is directed into the endosomal pathway. As endosomes mature into late endosomes and then lysosomes, the pH within endosomes reduces. This acidification leads to the liberation of the ligand from the complex. At this point, late endosomes undergo exocytosis, either recycling the ligand to the luminal site or liberating it to the abluminal site of the brain endothelium.^105,106^ Molecules that are not exocytosed are finally degraded in the highly acidic lysosomes.^105,106^ Villaseñor et al.^107^ conducted live imaging studies to elucidate the mechanisms involved in intracellular sorting after RMT in BECs. Their research indicates that intracellular tubules, which primarily contribute to RMT (Figure 2), are formed; endocytosed molecules not associated with tubules are more likely to be degraded in lysosomes and not exocytosed. Furthermore, they show that bivalent Fab fragments targeting TfR were more likely to be sorted to lysosomes compared with monovalent Fabs.^107^ Notably, this observation was consistent with other studies.^108,109^ The authors proposed that bivalency leads to receptor cross-linking and, thus, receptor clusters at the plasma/endosomal membrane.^107^ This, in turn, increases endocytic uptake into the BECs but decreases sorting into the narrow sorting tubules, possibly due to their larger geometrical size or reduced transmembrane diffusion rate.

### 2.5 Nose-to-Brain Delivery

Protein or peptide therapeutics can reach the CNS via intranasal administration, utilizing the olfactory or trigeminal pathway. These are anatomical connections between the nasal cavity and the brain, thereby circumventing the BBB. Therapeutics are taken up by olfactory epithelial cells and further endocytosed by olfactory sensory neurons, which project directly to the olfactory bulb in the CNS. In the case of the trigeminal pathway, intranasally administered proteins or peptides are endocytosed by the respiratory and/or olfactory epithelium and further delivered to the brainstem and/or olfactory bulb via trigeminal nerves, which innervate olfactory and respiratory mucosa.^110,111^ Although the exact molecular mechanisms of epithelial and neuronal uptake are still under debate, RMT and AMT have been utilized to improve the uptake into the brain following intranasal administration.

Of note, the formulation used to apply the macromolecules in the nasal cavity has to be adapted to the physiological environment of the nasal mucosa (pH 5.5–6.5 and osmolarity of 290–500 mOsm/kg) to avoid irritation and, thus, avoid excessive mucosal clearance and a decrease in bioavailability.^112^ Mucosal/mucociliary clearance is a self-clearing mechanism of the respiratory system through which molecules trapped in the mucus layer are transported to the nasopharynx due to ciliary movements on the nasal epithelia.^113^

## 3 Determining BBB Permeability

Overcoming or bypassing the BBB is a prerequisite for CNS-active therapeutic compounds to reach substantial exposure to their targets and, therefore, therapeutic activity.

During the preclinical phase, in particular, assessment of *in vitro* BBB permeability is crucial for validating a compound’s concentration in the CNS. There have been significant advancements in cell-based *in vitro* models of BBB permeability, which have been comprehensively reviewed.^114–117^ Here, we provide a short overview of the most common *in vitro* and *in vivo* methods for determining the BBB permeability.

A common approach for modeling the BBB *in vitro* is the use of transwell models, where BECs are grown on a filter membrane suspended in well insert of cell culture plates.^118^ The area above the well-insert simulates the apical side of BECs, while the space below represents the basolateral side. The test compound is introduced into the well-insert, and its concentration is measured over time in the basal chamber. Depending on the cell types used, e.g., human induced pluripotent stem cells (iPSCs), primary brain microvasculature endothelial cells, or immortalized cell lines such as human cerebral microvasculature endothelial cells/D3 (hCMEC/D3), transwell–BBB models exhibit variations in permeability due to differences in the integrity of the cell layer, varying expression levels of tight junctions, transporters, and receptors.^119–121^ To assess the BBB model’s paracellular integrity, the transepithelial/-endothelial electrical resistance (TEER)^122^ is used as a quality assessment. Coculturing BECs with primary PCs and ACs leads to a more realistic model and higher BBB integrity.

Transwell models, as static models, do not account for the influence of shear stress, especially on cell differentiation. Furthermore, the 2D format of the cell layer^123,124^ leads to phenotypes that less closely resemble those of the *in vivo* BBB.^125,126^ On the other hand, dynamic BBB models account for shear stress by utilizing pumps to simulate blood flow in the brain microvasculature.^127^ These dynamic models achieve higher TEER values and more realistic BBB properties. However, their suitability for high-throughput screenings (HTSs) and ease of use can be challenging.^115^ To enable HTS, microfluidic BBB models, generally composed of two perpendicular channels connected by a semipermeable membrane, have been developed.^128^ Additionally, organoids have emerged as a significant development in this area. In these cutting-edge techniques, spherical BBB models are used, where BECs, primary PCs, and primary ACs self-assemble in a 3D culture to closely resemble the *in vivo* composition. Direct interaction within all cell types results in a high *in vitro–in vivo* correlation. Furthermore, by choosing a suitable design, this approach offers the potential for high-throughput testing.^129–131^ All of these models differ in their ease of use and setup, throughput of screening, cost, and overall *in vitro–in vivo* correlation, as summarized in Table 2.

**Table 2:** *In Vitro* BBB Models Compared to Each Other^a^

model	ease of use/setting up	throughput	cost	*in vitro*–*in vivo* correlation^b^
monoculture transwell models^b^	+++++	+++++	+++++	+
coculture transwell models^b^	+++	++++	+++	++
monoculture dynamic models, microfluidic^c^	+++	+++	+	+++
coculture dynamic models, microfluidic^c^	+	++	-	++++
organoids	-	++	-	+++++

a The “+” or “-“ sign indicates whether the respective model is beneficial or disadvantageous, respectively, in comparison to the other within the same category.

b *In vitro*–*in vivo* correlation is highly dependent on the BEC-type used (highest TEER values achieved with iPSCs).

c Ease of setting up, scalability, and cost depend on whether the frame for the dynamic, microfluidic model is commercially acquired or self-made.

In approaches to directly determine compound exposure in animal models of a peripherally injected compound, brain tissues are harvested from either the whole brain or sections of interest and homogenized to extract the analyte. Subsequently, the analyte concentration is determined using a diverse set of methods, e.g., enzyme-linked immunosorbent assay (ELISA),^75,132^ radiolabeling methods,^133^ or liquid chromatography–tandem mass spectrometry (LC-MS/MS).^134,135^ To prevent false-positive detection of compounds in the blood vessel’s lumen, an intracardiac perfusion with saline buffer is performed before brain tissue is harvested. Despite the use of intracardiac perfusion, the analyte can still be bound or entrapped in the BECs, leading to an overestimation of brain exposure. More reliable results can be obtained using capillary depletion,^114^ a technique developed by Triguero et al.^136^ to remove most of the brain capillaries from the brain parenchyma. After mild homogenization (8–10 strokes with a glass homogenizer), the homogenate is centrifuged at 5400*g* for 15 min at 4 °C to separate the brain parenchyma from the brain vasculature, red blood cells, and brain nuclei.^136^ By quantifying the analyte in the brain parenchyma fraction and comparing it with that in the vasculature fraction, the method allows for quantification of the fraction of analyte crossing the BBB.

In many studies, cardiac perfusion is performed without using capillary depletion,^75,132–135^ which is associated with the risk of overestimation of BBB permeability. As an example, Pozzi and colleagues^137^ observed the distribution of an intranasally administered mAb in mice across various brain regions, including the olfactory bulb, hippocampus, cortex, brainstem, and cervical region of the spinal cord. They performed intracardiac perfusion, following brain section homogenization and subsequent histological examination via Western blot. However, fluorescence imaging of brain sections revealed that the mAb was predominantly confined within the blood vessels. This false-positive detection of the mAb in the brain regions could have been the result of inadequate intracardiac perfusion or due to binding of the mAb to endothelial cells (e.g., receptors) or its entrapment within BECs, underlining the necessity of capillary depletion.

Intracerebral microdialysis entails the insertion of a microdialysis probe with a semipermeable membrane into the brain regions of interest, allowing for the sampling of a substance for analysis.^138^ This approach offers several advantages, such as assessing concentrations in living animals, evaluating the concentration of therapeutics in the brain parenchyma, and sampling with low background due to the MW cutoff of the dialysis probe.^138^ While microdialysis has been successfully established to determine *in vivo* BBB permeability,^139^ there are many challenges to consider. The insertion of the microdialysis probe requires significant expertise, as it can cause BBB leakage^140^ and damage capillaries, leading to false-positive results. Consequently, it is recommended that permeability studies are initiated after a lag phase after inserting the probe, to avoid overestimation of BBB permeability due to microlesions.

To determine BBB permeability to substances *in vivo*, their concentrations are often assessed in the CSF^115^ and used to extrapolate to CNS bioavailability (Table 3). However, there is no general correlation between the concentration of macromolecules in the CSF and their concentration in the brain parenchyma or brain interstitial fluid, mainly due to a lack of general studies.^141^ The concentrations of administered drugs in the CNS can differ greatly between patients, even when subjected to the same intraventricular dosing protocol with the same drugs.^142^

In recent years, the assessment of BBB permeability to CNS-active compounds in patients has been conducted using positron emission tomography (PET),^143,144^ where a radiolabeled derivative of the compound of interest is detected in the brain after intravenous (i.v.) application.^145^ Mercier et al.^146^ used this technique to evaluate BBB permeability to minzasolmin, a small-molecule inhibitor of protein misfolding in PD. Minzasolmin’s volume of distribution in the brain was calculated to be 0.5 mL/cm^3^ at the steady state, corresponding to a concentration in the brain tissue of 50% of the corresponding plasma concentration, showing good penetration of minzasolmin across the BBB. In addition to its use to estimate the exposure of therapeutic compounds to the brain, PET has also been used to detect biomarkers for AD and PD in the brain. Interestingly, mAbs are an attractive option for detecting amyloid-β with high affinity and specificity, but they are hampered due to their low level of exposure in the brain. Therefore, more recent approaches use F­(ab)­2 fragments targeting amyloid-β, conjugated to a transferrin receptor antibody to enable RMT crossing the BBB.^147,148^

Comparing data from different *in vivo* approaches, *in vitro* models, and correlating *in vitro* with *in vivo* results can be challenging, as each model exhibits variations in properties associated with barrier integrity, paracellular permeability, expression levels of efflux pumps or receptors, and general measurement conditions, including shear stress levels. Consequently, it is recommended that BBB permeability to drugs is assessed using multiple orthogonal approaches and compared with benchmarking probes measured using the same assay.

**Table 3:** Methods for BBB Permeability Assessment *In Vivo*

method	description of the method	advantages	problems
CSF:serum ratio	Compare concentration in serum and in CSF (through lumbar puncture), sampled at the same time points, quantified by ELISA, radioimmunoassay (RIA), or LC-MS/MS.	easy sampling	indirect CNS bioavailability assessment
		widely used	no correlation between CSF and brain bioavailability
		allows assessment in patients	CSF–brain permeability depends on the type of analyte
		quantitative assessment	the method used defines accuracy (e.g., LC-MS/MS can verify intact therapeutic reaching the brain, whereas fluorescence microscopy cannot)
brain homogenates	Brain tissue of animal models is harvested and homogenized to determine the concentration of the analyte (e.g., by ELISA, RIA, or LC-MS/MS).	direct CNS bioavailability assessment	euthanasia of the animal
		widely used	requires capillary depletion and cardiac perfusion for reliable results
		comparison between different brain sections possible	no reliable reference values for BBB permeability to compare with
		quantitative assessment	
brain segments/slices	Brain slices of animal models are analyzed by fluorescence microscopy using a fluorescence-labeled analyte.	direct CNS bioavailability assessment	euthanasia of the animal
		comparison between different brain sections of interest possible	fluorophore can have an influence on the BBB permeability of the analyte
		bound or in BEC entrapped analytes can be directly observed	requires cardiac perfusion to avoid analytes trapped in the vessel’s lumen
			crucial to confirm that the entire analyte, not just the potentially degraded fluorophore, crosses the BBB
microdialysis	Semipermeable membrane dialysis probes are inserted into the brain of living animals. At time points of interest, a dialysate sample can be collected simultaneously with a plasma sample to determine the brain:plasma ratio. The samples can then be analyzed using LC-MS/MS or ELISA.	direct CNS bioavailability measurement	high surgical expertise required
		MW cutoff of the probe results in clean samples	inserting the probe is critical as it should not disrupt any capillaries, which could lead to BBB leakage
		comparison between different brain sections of interest possible	diffusion into the probe/through the semipermeable membrane following the concentration gradient is limited by setting of the concentration equilibrium
		assessments in living animals	
		dialysates from the exact same probe in the brain of the same animal can be collected at different time points	
		quantitative assessment	
positron emission tomography (PET) imaging	Radiolabeled analyte is administered to animals, and dynamic PET scans are performed immediately afterward to assess brain uptake. Imaging is complemented by *ex vivo* analysis (e.g., brain/plasma ratios, high-performance liquid chromatography (HPLC)) to quantify the intact parent compound in the brain. In humans, the radiolabeled analyte is administered peripherally, and PET scans are performed alongside arterial blood sampling to generate time–activity curves. BBB permeability is then evaluated by calculating parameters such as total distribution volume and brain influx rate, which indicate the extent and rate of brain penetration.	noninvasive, in-human method	duration of PET scanning dependent on the isotope’s half-life used
		quantitative assessment	high cost and expertise needed
		assessment in living animals	requires arterial blood sampling (invasive and technically demanding)

## 4 Enhancing BBB Permeability to Macromolecular Therapeutics: Current Approaches

Several general approaches for increasing BBB permeation, which mostly aim to increase the circulation half-life and general membrane permeability, have been established. There is a broad range of strategies, especially for peptides, including methylation, halogenation of aromatic residues,^149–151^ N-terminal acylation,^152^ modification of the peptide’s length, incorporation of noncanonical amino acids, and variation of stereochemistry.^153^ These modifications generally improve the pharmacokinetic challenges that peptides face, such as rapid metabolism and excretion, low plasma stability due to peptidase lability, and low distribution volumes due to reduced membrane permeability. For further details on how to improve the pharmacokinetic challenges of peptides, we recommend more comprehensive reviews.^154,155^ In the following section, we specifically highlight methods for increasing the BBB permeability to macromolecules using RMT, CMT, and AMT.

### 4.1 Peptide-Based BBB Vectors

Molecules termed BBB vectors can transport therapeutic molecules across the BBB through RMT or AMT, functioning as “molecular Trojan horses”. Among these vectors are antibodies and proteins, such as TfR-binding mAbs or single-chain Fab fragments,^108,156^ bispecific antibodies (e.g., targeting both the TfR and the therapeutic target^157,158^ ), endogenous soluble melanotransferrin (sMTf),^159^ or single-domain antibodies targeting α­(2,3)-sialoglycoprotein receptors on BECs.^160^ Peptides, being smaller in size and amenable to chemical synthesis via solid-phase peptide synthesis, can also function as BBB vectors. Peptide-based BBB vectors have been developed using various strategies, including peptide phage display on BEC monolayers *in vitro*, peripheral *in vivo* injection of phage libraries in mice, structural dissection of protein ligands for BEC surface receptors, and utilization of toxins or epitopes from pathogens known to penetrate the BBB (after removal of their toxic elements).

#### 4.1.1 Vectors with Unidentified Transport Mechanisms

In a phage display experiment, Yamaguchi and colleagues^161^ panned a library of phages displaying cyclic peptides against a hCMEC/D3 cell monolayer and identified a peptide, referred to as the SLS peptide, capable of traversing this barrier model. The phages displaying SLS were observed to cross the BBB in ICR mice more efficiently than a control phage lacking SLS.^161^ This was transferable to liposomes decorated with SLS, which showed increased permeability across the BBB in a cell model and ICR mice following i.v. injection of the SLS-liposomes.^161^ Due to sequence similarity between the SLS peptide and vitronectin, an integrin receptor ligand, the authors proposed a transport mechanism mediated by binding to integrin receptors and subsequent micropinocytosis. Interestingly, the BBB permeation of the SLS–phage increased when they were coincubated with other integrin receptor ligands, such as RGD peptide or fibrinogen.^161^ The authors proposed that the RGD peptide and fibrinogen could trigger a conformational change in integrins, transitioning them from the inactive or intermediate-affinity state to the active high-affinity conformation,^162^ potentially boosting the binding affinity of SLS–phage to integrins and thereby increasing transport efficacy. Based on the promising *in vitro* and *in vivo* results, the SLS peptide can facilitate the transport of macromolecules with a hydrodynamic size similar to or smaller than the M13 phage (MW 16.3 MDa^163^) across the BBB.

Li et al.^164^ developed a peptide called TGN peptide that exhibited BBB-permeability when displayed on phages *in vivo*. Moreover, experiments injecting poly­(ethylene glycol)-poly­(lactide-*co*-glycolic acid) (PEG-PLGA) NPs, either uncoated or TGN-coated (at 1:3 and 1:1 TGN-NP ratios) intravenously into nude mice showed a correlation between TGN peptide loading and accumulation in the brain.^164^ One hour after the injection of TGN-decorated NPs, the brain uptake of these NPs was about 2- to 3.5-fold higher than that of undecorated NPs, as determined by analyzing brain homogenates with HPLC. Later work^165^ demonstrated the efficacy of using the TGN peptide to target neurodegenerative diseases in an *in vivo* model of AD. Beyond neurodegenerative diseases, the TGN peptide was also found to be a useful tool for targeting gliomas within the CNS in an *in vivo* model.^166^

Oller-Salvia et al.^167^ developed peptidic versions of apamin, a protein in bee venom that inhibits Ca^2+^-dependent potassium channels, with the aim of retaining its ability to cross the BBB while eliminating its toxic effects. Among the peptides developed, MiniAp-1 exhibited increased BBB permeability in a monolayer transwell bovine BBB model compared with apamin. The authors proposed that MiniAp-1 used an active transcytosis mechanism to traverse the BBB model, as its BBB permeability decreased following exposure to low temperatures or the mitochondrial electron transport chain inhibitor potassium azide.^167^ Additionally, MiniAp-1 demonstrated the capacity to transport conjugated dopamine and iAβ5 peptide, an inhibitor of α-synuclein and β-amyloid self-assembly,^168,169^ across the barrier model. Further structural analysis led to the development of the cyclic MiniAp-4 peptide, which exhibited higher permeability across the bovine BBB model.^167^ MiniAp-4 demonstrated the ability to transport green fluorescent protein (GFP; MW 25 kDa) across a human cell-based BBB model.^167^ By applying *in vivo* confocal fluorescence imaging, the MiniAp-4–Cyanine5.5 conjugate showed higher accumulation in the brain than the control Cyanine5.5–cysteamine following i.v. injection into CD1 mice.^167^

MiniCTX3 and MiniCTX2 are BBB-penetrating peptides derived from chlorotoxin (CTX), a venomous peptide originating from the Giant Yellow Israeli scorpion.^170^ CTX itself was observed to enter the brain without disrupting the BBB structure *in vivo*.^171^ Díaz-Perlas et al.^170^ proposed an active transcytosis/transport pathway for CTX based on its capacity to cross a human cocultured BBB transwell model and its impermeability in a parallel artificial membrane permeation assay (PAMPA). Structural dissection of CTX led to the design of two cyclic peptides, MiniCTX2 and MiniCTX3.^170^ MiniCTX3 exhibited 2.5–3-fold higher permeability across the BBB model than the original CTX peptide.^170^ Further experiments by the same authors showed that MiniCTX2- and MiniCTX3-conjugated carboxyfluoroscein and gold NPs had significantly higher permeability through the human BBB model than that of CTX-conjugated carboxyfluoroscein and gold NPs.

#### 4.1.2 Vectors Using RMT Mechanisms

One of the well-known peptides that can penetrate the BBB is Angiopep-2, which is a ligand for LRP-1. Demeule et al.^172^ showed that it crosses the BBB both *in vitro* (coculture transwell model) and *in vivo* (in situ brain perfusion). Angiopep-2 demonstrated more efficient BBB transport than proteins known to enter the CNS, such as aprotinin and transferrin.^172^ Subsequently, the research group conjugated paclitaxel, a cancer chemotherapeutic,^173^ to Angiopep-2 and demonstrated that the resulting conjugate, named ANG1005, could penetrate the BBB *in vivo*, as determined by using an in situ brain perfusion test using brain homogenates.^174^ Moreover, it increased the survival of mice injected intracerebrally with glioblastoma cells. Encouraged by these results, ANG1005 was introduced into a phase II clinical trial for the treatment of patients with breast cancer exhibiting leptomeningeal carcinomatosis and recurrent brain metastases.^175^ Intravenous administration of ANG1005 resulted in benefits for patients with both CNS and systemic diseases. Patients with leptomeningeal carcinomatosis who were treated with ANG1005 exhibited higher survival rates compared with historical controls.^175^ An open-label phase III study to assess ANG1005 efficacy over common treatments for HER2-negative breast cancer patients with newly diagnosed leptomeningeal disease started at the end of 2023, with the first results expected at the end of 2024 (NCT03613181). However, the study’s sponsor, Angiochem Inc., has not yet published any results.^176^ Given these promising outcomes, it is plausible to explore whether Angiopep-2-conjugated macromolecules can treat neurodegenerative diseases.

In addition to various approaches targeting the TfR for CNS delivery,^156,158,177,178^ Singh et al.^179^ examined a potential endogenous ligand of LRP-1,^180^ sMTf. While membrane-bound MTf can transport iron through the cell membrane, sMTf was found to do so across the BBB,^181,182^ and can also be utilized as a vehicle to cross the BBB.^159,183^ However, its relatively large size (∼80 kDa^184^) may limit its utility as a CNS-targeted delivery compound. Consequently, Singh and colleagues^179^ assessed whether peptide fragments derived from sMTf could retain shuttling activity across the BBB. One fragment, referred to as the MTf peptide (MTfp), was shown to cross an *in vitro* bovine BBB model and the BBB of mice after i.v. injection of Cyanine.5-labeled MTfp, as determined by analyzing respective brain segments using 3D fluorescence microscopy. Additionally, MTfp-conjugated mAbs showed 2-fold higher uptake in the brain compared with unconjugated antibodies following i.v. injection into mice.^185^ In a mechanical hyperalgesia animal model, an interleukin-1 receptor agonist alone had no effect; however, when coupled with or ligated to MTfp or MTf, the agonist exhibited a reversal of mechanical hyperalgesia.^185^ Furthermore, Eyford et al.^186^ successfully used MTfp to deliver NADPH oxidase-specific siRNA through the BBB to attenuate ischemic stroke in mice. In the structural studies conducted by Singh et al.,^179^ MTfp exhibited a transient helical character, likely responsible for LRP-1 binding.

Mucopolysaccharidosis type 7 is a lysosomal storage disease of the CNS that is induced by the accumulation of glycosaminoglycans due to a deficiency in the lysosomal enzyme β-glucuronidase. To address this disease, Xia et al.^187^ developed the nonameric B6 peptide from a phage display screening against soluble human TfR. The B6 peptide was conjugated to adenoviruses, which were used as vectors to achieve transfection of BECs with β-glucuronidase, leading to 3.5-fold higher gene transfer compared with unmodified adenoviruses.^187^ Later, Liu et al.^188^ used the B6 peptide to increase BBB permeability to PEG-poly­(lactic acid) NPs encapsulating the neuroprotective NAP peptide. Using a transwell BBB model, they demonstrated a time-dependent cellular uptake of B6-conjugated NPs. The authors investigated the uptake mechanism and suggested RMT through TfR, as they observed energy-dependent and clathrin-mediated endocytosis.^188^ *In vivo* studies of B6-NPs indicated that B6 enables higher NP accumulation in the brain compared with unconjugated NP. In addition, B6 reduced the accumulation of the nanodrug delivery systems in the lung and spleen.^188^ Ultimately, behavioral analysis of an AD mouse model dosed with NAP formulated in B6-NPs showed a significant improvement in learning and memory function compared with AD control mice.^188^

In addition, RMT has been used to increase the nose-to-brain uptake of the neuroprotective NAP peptide. The peptide was encapsulated in poly­(ethylene glycol)-poly­(ε-caprolactone) NPs decorated with lactoferrin.^189^ Lactoferrin serves as a ligand for several receptors, collectively referred to as “lactoferrin receptors”, e.g., CD14, LRP-1, intelectin-1, Toll-like receptor 2/4, cytokine receptor 4, and heparan sulfate proteoglycans.^190^ Those receptors are expressed on BECs, neurons,^191^ and respiratory epithelial cells.^192^ Following nasal administration of lactoferrin-free and lactoferrin-conjugated NAP-NPs, the authors showed that lactoferrin conjugation leads to higher bioavailability of NAP-NPs in several brain regions (e.g., olfactory bulb, olfactory tract, cerebrum with the hippocampus removed, cerebellum, and hippocampus) in the first 8 h.

#### 4.1.3 Vectors Using AMT Mechanisms

Increasing the positive net charge of a macromolecule may enhance its uptake into the brain through AMT. This is especially utilized by so-called cell-penetrating peptides (CPPs), which constitute a diverse group of peptides showing mostly amphiphilic or highly cationic structures or the ability to form α-helices.^89,193–195^ The structural characteristics of CPPs enable them to traverse cell membranes through clathrin-dependent and -independent pathways.^193,196^ This ability has prompted extensive exploration of CPPs for their potential to overcome the BBB and serve as CNS targeting vectors,^197–200^ as well as their use to improve the nose-to-brain delivery of therapeutic compounds.^201^ However, it is important to note that macromolecules with a highly positive charge might interact strongly with negatively charged serum proteins such as albumin.^202^ This interaction would potentially impede the rate at which the macromolecule crosses the BBB.

Here, a few examples of molecules transported using AMT are described: The capsid protein of dengue virus type-2 (DEN2C) and specific peptide sequences derived from it, such as PepM and PepR, are recognized for their capacity to cross cell membranes and facilitate the translocation of nucleic acids into cells.^194,203,204^ Notably, the α-helical domains of DEN2C appear to play a role in this cell-penetrating property. Neves et al.^205^ categorized the four α-helices of DEN2C^206^ as PepH1–PepH4 and evaluated their potential to cross a monolayer BBB model. Among these peptides, radiolabeled PepH1 and PepH3 exhibited high BBB permeability and low membrane retention.^205^ In CD1 mice, PepH3 demonstrated a rapid brain uptake of 0.31% of the injected dose per gram of brain tissue (ID/g) after 5 min following i.v. injection. However, 1 h later, only 0.03% ID/g was detected, indicating rapid excretion from the brain.^205^ Based on this fast kinetics, the authors hypothesized an adsorptive-mediated transport/transcytosis mechanism.

The peptide derived from the transactivator of transcription (Tat) protein of HIV-1 ^207^ belongs to the highly cationic-charged CPP family. Schwarze and colleagues^208^ developed fusion proteins containing an N-terminal Tat peptide and β-galactosidase and observed a version labeled with fluorescein in the brains of mice after intraperitoneal (i.p.) injection; however, they did not observe fluorescein-labeled β-galactosidase alone. In a different study, Cao et al.^209^ observed a dose-dependent increase in the uptake of a fusion protein consisting of Bcl-xL (an endogenous antiapoptotic protein^210^) and Tat peptide after i.p. injection in the brains of mice, as determined via Western blot analysis of brain sections. This elevated level of peptides in the brain resulted in a reduced level of neuronal cell death after ischemic damage. In line with this, Shadmani et al.^211^ reported enhanced accumulation of Tat-decorated and methotrexate-loaded NPs in mice brains after i.v. injection. However, they also noted a dose-dependent increase in the hemolysis rates of erythrocytes by the Tat-conjugated NPs. Increased hemolytic activity was also seen in highly positively charged amphipathic antimicrobial peptides due to stabilization of transmembrane pores.^92,93^ Additionally, Stalmans et al.^197^ observed an accumulation of the Tat peptide in peripheral organs, especially in the liver. Thus, the selection of a CPP as a vector for BBB delivery should be approached with caution; furthermore, the mechanisms underlying these undesired effects require further detailed elucidation.

### 4.2 Glycosylation

An alternative strategy for delivering macromolecules into the CNS is glycosylation. This carrier-mediated transport (Figure 2)^20,212–214^ exploits glucose transporter type 1 (GLUT1). Among the various carrier systems at the BBB (see Table 1), GLUT1 stands out due to its 15–50 times higher capacity compared with other transporter systems,^215,216^ making it the most promising transporter for CNS delivery. Two approaches have been employed to facilitate the delivery of macromolecules to the brain using this method: (1) using nanodrug delivery systems decorated with glucose moieties and (2) directly conjugating macromolecules with sugar moieties, including glucose.^217^

#### 4.2.1 Using Glycosylated Drug Delivery Systems

Xie et al.^218^ explored different liposomes, where glucose is linked to cholesterol through PEGs of varying chain lengths. The PEG chain length is crucial for efficient exposure of the glucose molecules, as excessively long PEG chains are likely to be folded and might shield the glucose. Liposomes decorated with glucose via a PEG1000 linkage exhibited the highest *in vivo* uptake into the brain after i.v. injection in mice and in an *in vitro* coculture BBB model.^218^

Additionally, glucose–PEG-modified nanomicelles were used to transport 3D6 Fab fragments, targeting β-amyloid (Aβ), across the BBB in an AD mouse model.^219^ To confirm GLUT1-mediated transport, the authors showed a decreased brain uptake into primary rat BECs in the brain using the GLUT1 inhibitor phloretin.^219^ For recognition of glucose by GLUT1, the PEG chain is preferably connected to the C6 position of glucose.^220^ Following i.p. injection in mice, the native Fab exhibited a minimal uptake of approximately 0.07% ID/g into the brain, and nonglycosylated nanomicelles showed an uptake of 0.17% ID/g.^219^ In contrast, nanomicelles with 25% glucose-conjugated PEG chains demonstrated the highest accumulation in the brain at 2.93% ID/g. Additionally, glucose conjugation led to negligible accumulation in peripheral organs.^219^ Higher decoration percentages, specifically 50% and 100%, resulted in decreased uptake into the brain in these *in vivo* experiments. The authors propose that the increased avidity of the heavily glucose-loaded nanomicelles to GLUT1 likely leads to a greater retention within capillaries and decreased release into the brain parenchyma. Ultimately, the authors observed a 56% reduction in the formation of new Aβ plaques in AD mouse models.

Furthermore, various small molecules and nucleic acids targeting glioblastomas or other CNS targets have utilized glucose-modified nanodrug delivery systems.^221–224^

#### 4.2.2 Direct Glycosylation

In addition to using vehicles such as liposomes for delivery, peptides can be directly glycosylated or sugar-modified (e.g., lactose) to enhance BBB penetration. While glucose-decorated nanodelivery systems, such as liposomes, facilitate BBB crossing through CMT,^220^ directly glycosylated peptides (glycopeptides, GP) employ other pathways. For GPs, it is suggested that higher uptake into the brain occurs through a phenomenon known as “membrane hopping”, whereby the GP adopts a helical conformation near the membrane of BECs and a random coil conformation in the extracellular environment^225^ (Figure 3). This rapid change in conformation and the direct interaction with the luminal membrane’s glycocalyx provide the peptide with the opportunity to undergo transcytosis via AMT^225^ or RMT.

**Figure 3:**
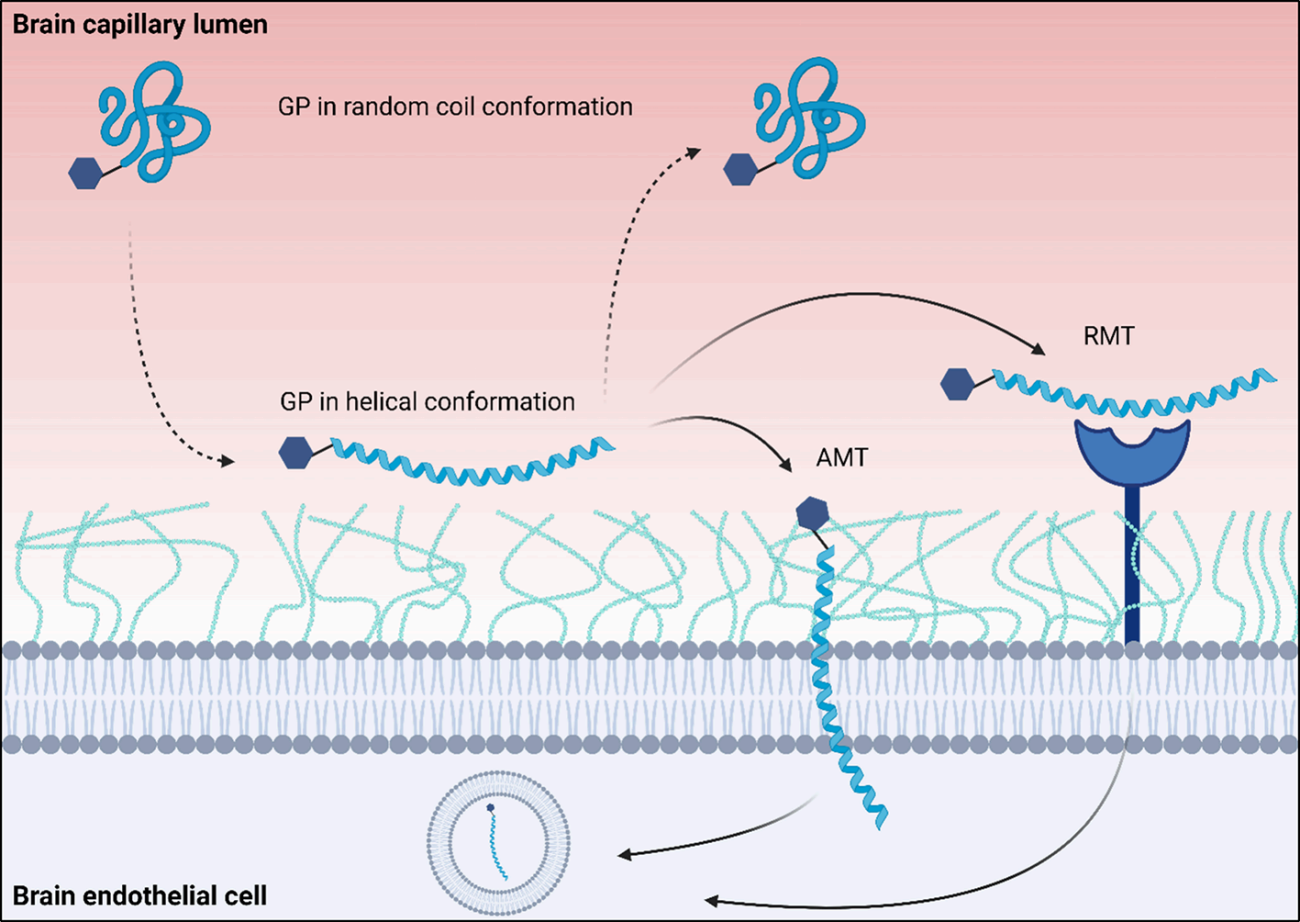
Schematic illustration of “membrane hopping”. The glycopeptide (GP) hops from one membrane site to the other, effectively searching for its receptor for RMT or increasing the possibility of AMT through an interaction with the glycocalyx. Created in BioRender (https://BioRender.com/ivhl6kr).

Apostol and colleagues^226^ examined the impact of sugar modification on the neuroprotective pituitary adenylate cyclase-activating polypeptide (PACAP) for traumatic brain injury (TBI)-induced central deficits and PD in respective mouse models.^227,228^ PACAP is a naturally occurring peptide released by pituitary glands. Apart from its diverse effects, such as regulating ion concentration in the kidneys,^229^ PACAP is known for promoting cell survival and healing, particularly within the CNS.^230,231^ The role of PACAP has been extensively demonstrated in various animal models, including those associated with AD^232–234^ and PD.^235–237^ As PACAP is known for its poor stability *in vivo* and limited BBB penetration,^238–240^ Apostol et al. modified PACAP1–38 with carbohydrate molecules to minimize these limitations.^226^ The lactose-modified PACAP derivative demonstrated increased CSF concentration, improved sleep-wake behavior, motor skills, and cognition in the TBI mouse model, as well as enhanced motor function and attenuated tyrosine hydroxylase-positive neuronal cell death in the substantia nigra in the PD mouse model.^226^

Varamini and co-workers^241^ developed a lactose-conjugated version of endomorphine-1, demonstrating improved efficacy compared with the native peptide in a rat model of chronic pain. Interestingly, the lactose derivative exhibited enhanced oral absorption and pain relief, achieving a potency comparable to that of morphine after oral administration. However, the modification with lactose resulted in a reduction in receptor binding affinity and agonist activity at the μ-opioid receptors.^241^

GP opioids have demonstrated efficacy not only in antinociception but also in PD animal models for treating levodopa-induced dyskinesia.^242,243^ Prolonged treatment of PD patients with levodopa results in dyskinesia, accompanied by increased levels of opioid peptides and their mRNAs in the striatum.^244,245^ Thus, opioid transmission is elevated, as either a compensatory response or a contributing factor to dyskinesia. Mabrouk and colleagues^246^ showed that selective μ-opioid receptor antagonism and δ-opioid receptor agonism lead to a potent dopamine denervation treatment; their findings are supported by several other studies.^247–249^ In alignment with these findings, Yue and co-workers^250^ investigated the ability of a GP opioid agonist, MMP-2200, to penetrate the BBB and accumulate at its active site in the brain, the dorsolateral striatum (DLS). Their experiments in adult male Sprague–Dawley rats, involving i.p. administration of the peptide, demonstrated the accumulation of MMP-2200 in the DLS, as determined by microdialysis, indicating efficient penetration of the BBB. Moreover, the GP reduced hyperkinetic movements induced by the dopamine agonist apomorphine in dopamine-depleted rats following systemic administration.^250^ This effect on dyskinesia was also observed in the context of dopamine receptor 2-like agonist-induced dyskinesia.^251^ Interestingly, while the nonglycosylated counterpart of MMP-2200 exhibited similar receptor-binding affinities to MMP-2200, glycosylation led to a 10-fold increase in potency *in vivo* after i.v. administration.^252^

Despite impairment in receptor binding affinity and activity, sugar-modified peptides may exhibit higher CNS efficacy and potency *in vivo* compared with those of peptides without these modifications. In line with this, Craig and colleagues^253^ reported greater *in vivo* efficacy of the GPs over their nonglycosylated counterparts, which is attributed to higher BBB permeability and increased serum stability due to hindrance of protease or peptidase binding to the peptide.^254,255^ A “glyco-scan” approach, as conducted by Pedersen et al.,^256^ will help determine the site-dependent effects of glycosylation on a specific peptide, including its impact on plasma half-life and receptor binding.

## 5 Macromolecular Therapeutics for Neurodegenerative Diseases: Current Insights from Preclinical and Clinical Studies

Extensive efforts have been made in recent years to develop disease-modifying macromolecules for the treatment of neurodegenerative diseases (Table 4). Antibodies are the most frequently investigated macromolecules for these disease indications; many are currently in preclinical and clinical development, and three have been approved (one of which has been discontinued). This is remarkable considering the above-discussed characteristics of BBB permeability. In this section, we summarize the macromolecular therapeutics that are currently in development for neurodegenerative diseases such as AD and PD and highlight clinical evidence of their efficacy. Furthermore, we focus on macromolecular therapeutics and BBB-penetrating mechanisms and discuss the available data on their pharmacokinetic and pharmacodynamic behaviors.

**Table 4:** Macromolecular Therapeutics in Clinical and Preclinical Development for Neurodegenerative Diseases

neurodegenerative disease	compound name	macromolecule type	target(s)	stage of clinical development	beneficial outcomes	negative outcomes	BBB permeability
AD	aducanumab	human mAb (IgG1 type)	aggregated-soluble and - insoluble Aβ	FDA approved (discontinued)	removal of Aβ plaques (determined by ^18^F-florbetapir PET imaging)	ARIA-related microhemorrhages	brain:plasma AUC ratio of 1.3% in mice determined by ELISA
						no evidence of therapeutic benefit in clinic	
	lecanemab	humanized mAb (IgG1 type)	Aβ protofibrils, a soluble Aβ aggregate	FDA approved	reduction of amyloid burden	ARIA-related edema or effusions	brain penetration of 0.24 ± 0.12% ID/g in mice determined by ELISA
					moderate effect on cognition and function loss in early-stage AD patients^283^		
	donanemab	humanized mAb (IgG1 type)	N-terminally truncated and pyroglutamate-modified Aβ (soluble oligomers)	FDA approved	reduction of amyloid burden	ARIA-related edema or effusions	CSF:serum concentration of 0.2% determined by ELISA
					significant deceleration of clinical AD progression in early-stage AD patients		
	bapineuzumab	humanized mAb (from mouse)	soluble Aβ monomers and aggregates	phase III – terminated	improved key biomarkers of AD (Aβ brain plaque determined by Pittsburgh compound B PET imaging)	ARIA-related microhemorrhages	semiquantitative determination of brain accumulation via ^125^I-radiolabeling (no reference declared/compared to plasma pharmacokinetic features)
						lack of clinically detectable improvement	
	davunetide	peptide (8-mer, linear)	neuroprotective properties	mouse model (for AD)	improved Aβ brain clearance and cognitive function in an AD mouse model	no clinical advantage in tau-pathological diseases including supranuclear palsy	CSF:plasma *C*_max_ ratio of 4–5% for intranasal or i.v. administration (determined by LC–MS/MS of samples of CSF and plasma of treated AD patients)
				phase III (tau-pathological diseases)	disassembly of preformed Aβ plaques in *in vitro* assays		
	TFP5	peptide (34-mer, linear)	Cdk5/p25	preclinical - AD mouse model	reduction/prevention of Aβ plaque formation	(not tested in humans yet)	semiquantitative determination of brain accumulation (hippocampus, cortex, and cerebellum) via fluorescence imaging
					reduction of tau phosphorylation		
					reduction of inflammation and gliosis		
PD	prasinezumab	humanized mAb (IgG1 type)	C-terminus of aggregated aSyn	phase IIb (running until probably 2026)	protection against synaptic loss and gliosis in animal models	lack of efficacy in increasing dopamine transporter levels in brain (as a biomarker – determined by ^123^I-ioflupane single-photon-emission computed tomography)	quantitative analysis of the antibody's titer in brain homogenates compared to in plasma via ELISA (in mice)
					reduction of intracellular aSyn pathology and cell-to-cell transmission of aSyn in an animal model	no clinical efficacy in slowing PD progression in humans	CSF:plasma *C*_max_ ratio of 0.3% for i.v. administration determined by sandwich ECL (in humans)
					reduction of serum aSyn as a clinical biomarker in humans		
		
	cinpanemab	human mAb (IgG1 type)	N-terminus of aggregated aSyn	phase II – terminated	delay in the onset of paralysis symptoms, reduction of truncated aSyn, and improvements in motor function in preclinical studies	no improvement in prevention of dopamine transporter loss in the brains of humans	CSF:plasma *C*_max_ ratio of 0.128–0.250% for i.v. administration to healthy volunteers and 0.273–0.559% for PD patients
							determined by SEC followed by ELISA
					decreased dopamine transporter loss in a PD mouse model	no significant differences were observed in motor and nonmotor dysfunction	
	AmyP53	peptide (12-mer, linear)	gangliosides GM1 and GM3	*in vitro* studies	prevents binding of amyloid proteins (aSyn and Aβ) to GM1 and GM3 and, thus, reduces amyloid pore formation	(not tested in humans yet)	BBB penetration after intranasal and i.v. injection to rats (determined by liquid chromatography–mass spectrometry (LC–MS) measurement of brain homogenates)
							coculture-based transwell BBB assay – concentrations determined by LC–MS
ALS	α-miSOD1	human mAb (IgG1 type)	human misfolded SOD1	preclinical, animal models (SOD1-G93A and SOD1-G37R tg mice)	66% reduction of misfolded SOD1 in B6SJL-SOD1-G93A tg mice after i.p. therapy	(not tested in humans yet)	(not tested yet but crucial to improve efficacy in humans)
					reduction of micro- and astrogliosis in an animal model		
					improvement in gait abnormalities and movement patterns of hindlimbs		
					delay onset of disease and prolonging of survival		
	E6	mouse mAb (IgG2a type)	RRM1 domain of TDP43	preclincal, animal models (TDP43-A315T tg mice)	reduction of NF-κB-induced microglial hyperactivation	(not tested in humans yet)	trapped in blood vessels after intranasal administration and slight distribution into some CNS regions determined by fluorescence imaging in TDP43-A315T tg mice
					reduction of cytoplasmatic TDP43 and pathogenic p65 NF-κB		
	tofersen	antisense oligonucleotide (RNase H1-dependent mode of action)	mRNA of SOD1-G93A	FDA approved	reduction of CSF SOD1 and plasma neurofilament light chain concentration	intrathecal administration	(not tested because intrathecal administration)
					significant efficacy in patients at trial entry (possible efficacy in presymptomatic SOD1 variant carriers)	no improvement in slowing vital capacity and in hand-held dynamometry (expect patients at trial entry)	

### 5.1 Alzheimer’s Disease

AD is a progressive neurodegenerative condition characterized by memory loss and executive function deterioration, along with personality changes.^257^ The primary symptom of memory loss stems from progressive synapse loss and neuronal atrophy, starting primarily in the hippocampus.^258^ Early-onset AD is characterized by genetic variations in disease-associated genes (e.g., PSEN1/2 or βAPP), while the exact cause or trigger of late-onset AD remains largely unknown.^259^ The amyloid hypothesis is the prevailing and widely accepted theory for the possible AD pathomechanism (Figure 4). In a healthy state, the Aβ precursor protein (APP), a transmembrane protein found on neurons’ plasma membranes, is hydrolyzed by α-secretases and γ-secretases to nontoxic, monomeric, and soluble fragments (p3 and the ectodomain APPsα). This is the so-called nonamyloidogenic pathway, and monomeric Aβ is found in plasma and the CNS, where it plays a role in antimicrobial and synaptic functions, memory, and recovery from brain injury.^260^ In the disease state, APP is abnormally processed by β-secretases, particularly β-secretase 1 (BACE-1), and γ-secretases, leading to the production of insoluble and toxic Aβ (amyloidogenic pathway).^261^ Aβ1–40 has been shown to form pores in the neuronal membrane, resulting in a toxic increase in intracellular Ca^2+^ ions.^262^ Additionally, the amyloidogenic pathway leads to Aβ aggregating into oligomers and so-called extracellular plaques, which results in synaptic dysfunction and loss^263,264^ via excitotoxicity, oxidative stress, and mitochondrial dysfunction.^265^ Gene mutations, for example, at the cleavage site of APP^259^ or involving BACE-1 (more specifically, the major components of BACE-1: PSEN-1 and -2),^265^ mainly contribute to the formation of insoluble Aβ.

**Figure 4:**
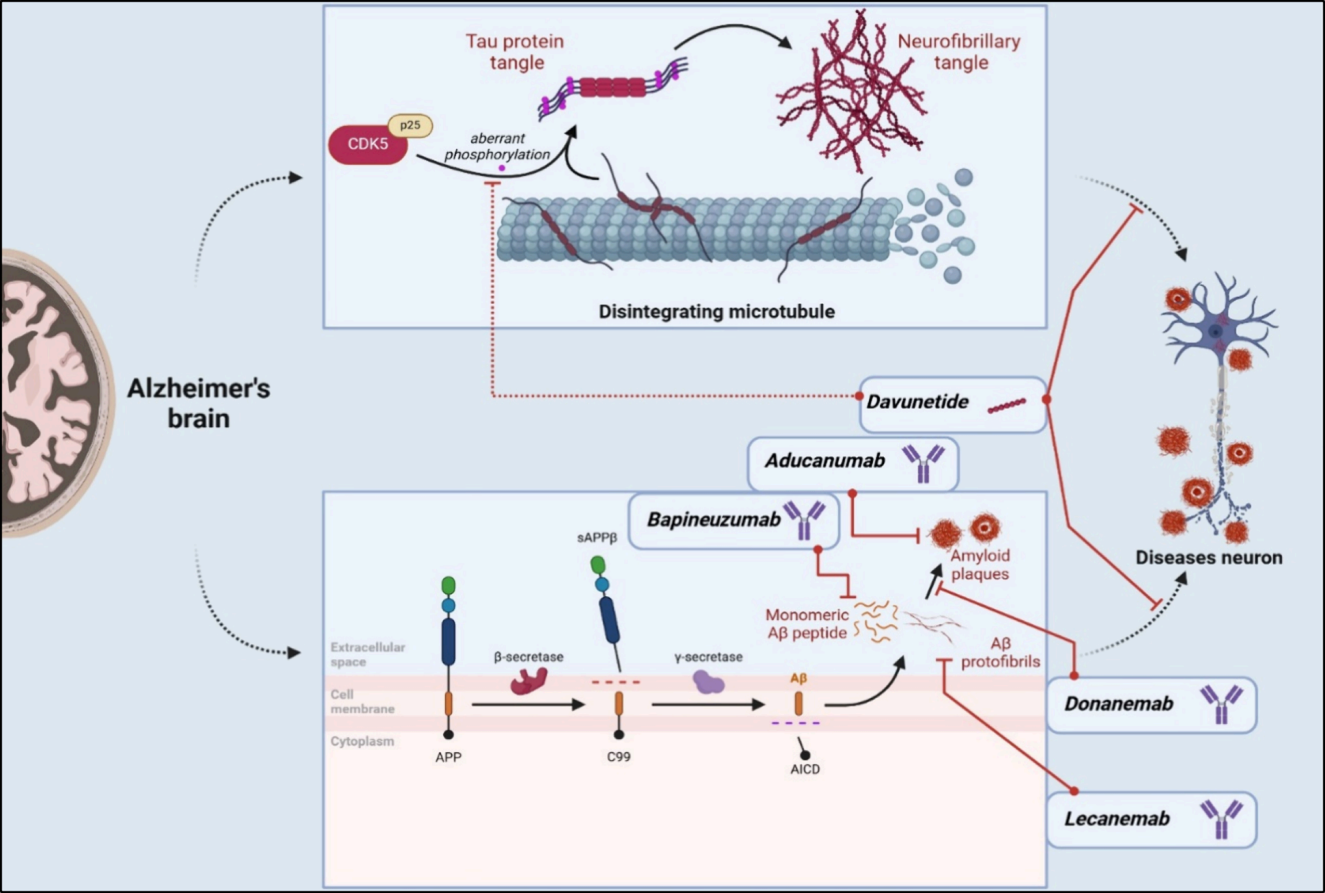
Mechanism of action of promising macromolecular therapeutics in AD. Created in BioRender.

Tau proteins also play a role in AD’s pathogenesis.^266^ Tau proteins stabilize microtubules, while hyperphosphorylated tau proteins are associated with a higher tendency to aggregate, forming fibrils in the cytoplasm of neurons (neurofibrillary tangles), ultimately leading to neurodegeneration.^267^ Additionally, the loss of tau protein function contributes to deficiencies in axonal transport.^268^

In line with the amyloid hypothesis, various strategies have been pursued to develop mAbs targeting Aβ oligomers and plaques.^269^ The first mAb to receive FDA approval for this purpose is aducanumab^270^ (now discontinued). This human anti-Aβ mAb specifically targeted aggregated Aβ and Aβ oligomers, with limited affinity for Aβ monomers.^271,272^ After i.p. injection of 2576 transgenic (tg) mice, a brain/plasma area under the curve (AUC) ratio of 1.3%^75^ was observed, implying relevant penetration into the brain. This result was obtained through ELISA experiments using brain homogenates without performing capillary depletion; therefore, the high AUC ratio might be a result of incomplete washout of cerebral blood volume,^273^ with aducanumab getting sequestered in the brain vessels rather than entering the brain. However, aducanumab is reported to inhibit Aβ neurotoxicity in 2576 tg and βAPP/PSEN1 tg AD mouse models, leading to a reduction in plaques, toxic Ca^2+^ signaling, and hyperphosphorylated pathogenic proteins. Furthermore, it enhanced phagocytosis by stimulating microglia.^269,274^ By utilizing PET imaging techniques, aducanumab was shown to remove amyloid plaques in the phase Ib PRIME clinical trial.^75^

However, there is a big scientific controversy due to the lack of significant benefits for AD patients.^275,276^ While aducanumab reduces Aβ levels in the brain, there is no conclusive evidence of its impact on the clinical symptoms of AD. On the other hand, aducanumab is associated with the risk of amyloid-related imaging abnormalities (ARIA)-related microhemorrhages.^276,277^ Consequently, the FDA called for an additional assessment of the tolerability, safety, and efficacy of aducanumab. It was ultimately terminated in July 2024 due to discontinuation by the sponsor (Biogen).^278^

Lecanemab (or BAN2401), also recently approved by the FDA, is a humanized mAb that selectively binds to Aβ protofibrils, which are soluble Aβ aggregates.^279^ In a phase III clinical trial, amyloid PET showed that lecanemab removes insoluble amyloid plaques.^280^ Seven days after i.p. injection of a murine version of BAN2401 (mAb158) into ArcSwe tg mice, an AD mouse model, 0.2% of the antibody reached the brains (quantified by ELISA of mice’s brain homogenates^279^). Although the authors did not evaluate the BBB permeation mechanism of the antibody, the slow kinetics of brain penetration (0.1% blood/plasma on day 1 and 0.2% blood/plasma on day 7) concur with BBB penetration through extracellular pathways. In line with this conclusion, single-photon emission computed tomography (SPECT) did not detect any ^125^I-radiolabeled mAb158 in the brain of ArcSwe tg mice 3 days after i.v. injection, but it did 6 days postinjection.^281^ SPECT analysis showed retention of the antibody in the brain ventricles 14 and 27 days postinjection. A maximum brain penetration of 0.24 ± 0.12% ID/g was documented in this study, which is only slightly higher than the commonly used reference value of 0.1% ID/g for CNS-active mAbs.^78^

The FDA-approved humanized mAb donanemab specifically targets N-terminally truncated and pyroglutamate-modified Aβ, which forms soluble and neurotoxic oligomers/amyloid plaques.^282^ In a phase Ib study of AD patients, 0.2% of the serum concentration was detected in the CSF 72 h after i.v. administration (determined by ELISA).^283^ To the best of our knowledge, no other studies have been conducted to assess the mAb’s BBB permeability. However, given the slow kinetics of its transport (0.2% of serum concentration after 3 days), we propose that the antibody enters the brain via extracellular pathways. In phase I,^283^ II,^284^ and III^285^ studies, donanemab was shown to remove amyloid plaques in early-stage AD patients’ brains, as determined using ^18^F-florbetapir (a common radiotracer for amyloid plaques^286^) PET, assuming sufficient BBB permeability. Encouraged by the positive results of the clinical progression of early-stage AD patients in a phase III study,^285^ the FDA approved donanemab for adults with early symptomatic AD.

Bapineuzumab is a humanized anti-Aβ mAb designed to target both soluble Aβ monomers and Aβ aggregates, with specificity for the helical conformation of the Aβ peptide’s N-terminus.^287^ While the exact mechanism of transport across the BBB was not investigated, Bard and colleagues^288^ observed the accumulation of a murine, ^125^I-labeled derivative of bapineuzumab in human APP tg mice’s hippocampus following i.v. injection. The maximum level of bapineuzumab in the mice’s brains was reached after 14 days and remained stable for over 10 days. This observation in mice, combined with the antibody’s extended half-life in humans, suggests that it may enter the brain via extracellular pathways. By assessing cortical fibrillary Aβ using its marker ^11^C-PiB (carbon-11-labeled Pittsburgh compound B) in PET scans, bapineuzumab demonstrated a reduction in Aβ accumulation in the brains of AD patients.^289^ However, bapineuzumab failed to show significant cognitive improvements in both ApoE4 carriers and noncarriers in the first two phase III trials.^290,291^ Bapineuzumab exhibited generally mild adverse events and favorable pharmacokinetic properties in a phase I clinical trial, with a half-life ranging from 21 to 26 days,^292^ while in later phase III studies, a high rate of side effects associated with ARIA was observed.^291^

More than 10 mAbs targeting Aβ have shown similar disappointing results in clinical trials.^293,294^ Solanezumab, for example, demonstrated increased brain clearance of the Aβ1–42 peptide in an APP tg mouse model^295,296^ and showed high target binding in the CSF.^297^ However, the mAb failed to show any improvements in the cognitive or motor functions of AD patients in two phase III trials,^297,298^ as well as in early application in patients with high genetic risk who started treatment before the onset of the first symptoms of cognitive decline.^299^

Davunetide is an eight-amino-acid-long peptide derived from the activity-dependent neuroprotective protein (ADNP), which was under investigation in phase III trials for the treatment of progressive supranuclear palsy, a neurodegenerative brain disease of the basal ganglia.^300^ It has been shown to reduce *N*-methyl-D-aspartate (NMDA) toxicity, Aβ toxicity, and ApoE deficiency^301,302^ in *in vitro* and animal models. The first *in vitro* results indicated that it might have benefits in AD as it inhibits Aβ aggregation, disassembles preformed Aβ aggregates,^303^ and reduces tau aggregation.^304^ In an AD mouse model (APP, PS-1, and tau mutants), intranasally administered davunetide led to a reduction in the levels of Aβ1–40 and Aβ1–42 in the brain,^305^ and in mouse and rat AD models, intranasal administration of the peptide reduced AD-related symptoms.^306,307^ In a phase I clinical trial, the peptide was administered intranasally, allowing it to access the brain parenchyma and CSF.^308,309^ In addition to the rapid increase in CSF exposure in AD patients following intranasal administration, i.v. davunetide administration exhibited similar CSF/plasma C_max_ ratios of 4–5%, as determined using LC-MS/MS,^310^ showing BBB permeability. Unfortunately, davunetide was not evaluated further to assess its efficacy in AD treatment, most likely due to disappointing outcomes for other indications of clinical trials. Multiple phase II/III clinical trials involving progressive supranuclear palsy patients treated with davunetide did not reveal any significant benefits,^300^ similar to studies focused on tau-pathological diseases, which also failed to demonstrate any therapeutic advantages for patients.^310,311^ Interestingly, sex-specific memory-enhancing and anxiolytic effects in prodromal AD have been identified in a more recent analysis, offering hope of a beneficial effect in women.^312^

### 5.2 Parkinson's Disease

PD is a neurodegenerative disease that affects 10 million people worldwide. Symptoms of PD include tremors and muscle rigidity, as well as nonmotor symptoms such as sleep or autonomic dysfunction.^313,314^

The exact pathogenesis is not fully understood,^10^ but dopaminergic neurons in the substantia nigra are primarily affected^315^ (Figure 5). Misfolded α-synuclein (aSyn) fibrils in the cytoplasm of neurons form structures known as Lewy bodies/neurites.^316^ Their accumulation ultimately leads to cell apoptosis.^317,318^ Under physiological conditions, aSyn maintains a dynamic equilibrium between a cytosolic and membrane-bound form, adopting either unfolded and monomeric/tetrameric or α-helical structures.^319,320^ Under pathophysiological conditions, for example, due to Ser129 phosphorylation, aSyn forms β-sheets, which aggregate and associate with Lewy bodies.^321,322^ Soluble oligomers of aSyn have been reported to exhibit toxicity due to pore formation in the neuronal membrane, which act as Ca^2+^ channels^323^ and lead to increased cytoplasmic Ca^2+^ levels and subsequent Ca^2+^ toxicity and cell death.^324–328^ Specifically, the interaction of aSyn and other amyloid proteins with gangliosides, specialized glycolipids in lipid raft domains of membranes,^329^ appears to initiate the formation of these toxic pores.^330,331^

**Figure 5:**
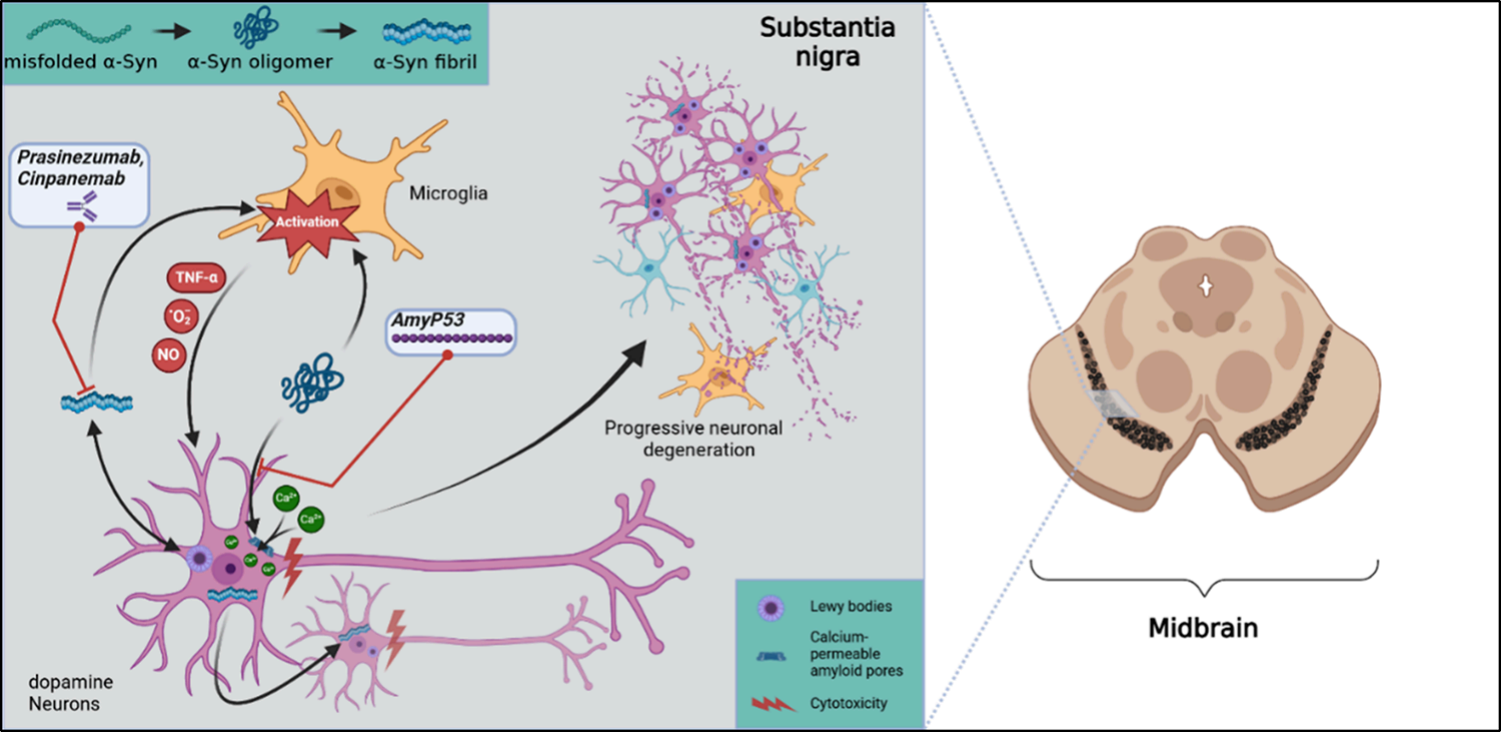
Mechanism of action of promising macromolecular therapeutics in PD. Created in BioRender.

Interestingly, during disease progression, Lewy bodies were shown to spread from one brain region to another via internalization of misfolded aSyn.^332–335^ Consequently, the clearance of extracytosolic and/or membrane-bound aSyn is believed to diminish aSyn propagation and pathology, thereby slowing down the progression of PD. Therefore, most therapeutics in development for PD treatment target extracellular aSyn oligomers.^336^

Serum aSyn concentration, which shows a 250–400× higher level than aSyn in CSF, moderately correlates with motor severity in patients with early PD^337^ and is therefore used as a biomarker, as aggregated aSyn concentrations in CSF are too low to be detected.^338^

Prasinezumab is a humanized mAb targeting the C-terminus of aggregated aSyn. Its murine derivative, 9E4, demonstrated therapeutic potential in preclinical trials by improving memory, learning, and motor deficits in aSyn tg mice.^132^ To assess its BBB permeability, the 9E4 level following administration of a single i.v. dose to aSyn tg mice was assessed in brain homogenates after 3, 14, and 30 days.^132^ On day 3 postinjection, the concentration was highest in plasma. On days 14 and 30, higher concentrations were observed in the brain and were detectable in the CSF, suggesting extracellular pathways as a transport route. In a phase I clinical trial,^339^ prasinezumab showed an increased concentration in the CSF (around 0.3% relative to serum) in a dose-dependent manner. The mAb effectively bound and cleared serum aSyn. However, prasinezumab did not demonstrate any advantages in slowing the progression of PD in a phase II study, nor did it have an impact on dopamine transporter levels following ^123^I-ioflupane (a radiotracer for imaging dopamine transporters) SPECT.^340^ A recent subgroup analysis of the PASEDENA study showed a beneficial effect of prasinezumab treatment for patients with rapid disease progression.^341^ However, it is under investigation in a phase IIb trial sponsored by Roche to assess safety and effectiveness in PD patients, with results expected by the end of 2026 (NCT04777331).

Cinpanemab (BIIB054) is a mAb that targets the N-terminus of aggregated aSyn fibrils with an 800-fold higher affinity for the aggregated form of aSyn compared with the monomeric form.^342^ Phase I studies showed a CSF-to-serum ratio of the intravenously administered antibody from 0.13% to 0.56% and BIIB054-aSyn complexes in plasma.^338^ Unfortunately, neither the BBB permeability nor the transport mechanism was investigated further. A phase II study of the antibody was terminated after week 72 of interim analysis due to a lack of efficacy.^343^ No significant differences were observed in motor and nonmotor dysfunctions assessed by the Movement Disorder Society-sponsored revision of the Unified Parkinson’s Disease Rating Scale (MDS-UPDRS).^344^ Disappointingly, results from dopamine transporter analysis by SPECT also yielded unsatisfactory outcomes.^343^ In contrast, a murine derivative of cinpanemab, chBIIB054, demonstrated a delay in the onset of paralysis symptoms following weekly administration to PD mice inoculated with preformed aSyn fibrils via intrastriatal injection.^342^ The studies also recorded a reduction in truncated aSyn levels in the contralateral cortex and improvements in motor function. Additionally, treatment with BIIB054 resulted in a decrease in dopamine transporter loss, measured by quantitative immunoblot analysis, in these mice.

AmyP53 (α-syn/HH) is a peptide that is currently in the preclinical phase of development. The peptide is derived from the binding sequence of aSyn and Aβ to gangliosides GM1 and GM3,^135,345^ showing reduced assembly of amyloid pores in neuronal cells after treatment with PD-associated mutant forms of aSyn and Aβ1–42.^346^ In *in vivo* rat models, 0.2% and 1.57% of intravenously injected and intranasally administered AmyP53 reached the brain, respectively.^135^ Experiments using a transwell-BBB model demonstrated transport effectiveness similar to the CPPs SynB3 and SynB5 through bEnd.3 cells (BECs from BALB/c mice) cocultured with cortex ACs.^135^ Moreover, AmyP53 exhibited biological activity by inhibiting Ca^2+^ influx through Aβ1–42.^135^ The peptide’s overall positive charge and its membrane-interaction capability suggest an uptake via AMT. As the peptide is partially derived from the LRP-1 ligand Aβ,^347^ transport across the BBB by RMT should also be considered.

### 5.3 Amyotrophic Lateral Sclerosis

In ALS, a degenerative motor neuron disease, motor neurons in the motor cortex, brain stem, and spinal cord are predominantly affected by degeneration.^348^ Its symptoms encompass progressive weakness, skeletal muscle atrophy, and, eventually, paralysis, leading to death within 3–5 years from the onset of the disease.^348^ Its pathology is not fully understood, although proteinopathies seem to play a significant role.

Misfolding of superoxide dismutase 1 (SOD1) is caused by mutations and possibly other, yet unknown triggers.^349,350^ Misfolding of SOD1 results in its hyperactivity, leading to inhibition of fast axon transport via activation of the p38-MAPK pathway,^350^ aggregation within the cells,^351^ oxidative and ER stress,^352^ mitochondrial dysfunction,^353^ and/or prion-like propagation,^354^ consequently leading to cytotoxicity.^355^ Therefore, the clearance of hyperactive SOD1 is a potential therapeutic concept. Additionally, TAR DNA-binding protein 43 (TDP43) contributes to ALS pathology. Under normal conditions, TDP43 plays a role in transcription repression and exon skipping.^356^ However, in pathological states, misfolded TDP43 is expelled from nuclei, leading to the formation of ubiquitinated inclusion bodies in the cytoplasm. The interaction between TDP43 and NF-κB leads to the coactivation of NF-κB’s proinflammatory and pathological pathways, such as microglia-mediated neurotoxicity (microgliosis).^357^ This results in the loss of protein function, ultimately causing cell death.^358,359^ While treatments are mostly developed with the objective of reducing degeneration in the spinal cord, it is important to mention that the degenerative process also affects motor neurons in the brain. For further insights into ALS pathology, we refer to comprehensive reviews on this area.^348,360–362^

α-miSOD1 is a human mAb specifically targeting human misfolded SOD1.^363^ Studies have demonstrated that intracerebroventricular injection of a murine chimeric derivative of the mAb into SOD1 tg mice results in clearance of SOD1 aggregates and a significant improvement in gait abnormalities and movement patterns of the hindlimbs.^363^ To further evaluate uptake into the spinal cord, the α-miSOD1 mAb was intraperitoneally injected into human SOD1 tg mice, exhibiting slowly progressing motor deficits.^363,364^ Twenty days postinjection, 0.4% of the plasma α-miSOD1 reached the spinal cord. Treatment of these mice with the antibody led to a delayed onset of the disease (by 49 days) and prolonged survival (by 59 days) compared to untreated control mice. Furthermore, a significant increase in the weight of hindlimb muscles and the preservation of 50% of motor neurons were observed.^363^ The authors suggested microglial phagocytosis of misfolded SOD1/α-miSOD1-mAb complexes as the main mode of action, where the mAb's Fc glycosylation is important for phagocytosis-inducing Fcγ receptor binding to microglia.^365,366^ Although it has been proposed that antibodies such as α-miSOD1 can penetrate the CNS due to impairments in the blood–spinal cord barrier in ALS patients and animal models,^363^ the exact relationship between blood–spinal cord barrier disruption and motor neuron degeneration remains unknown.^367^ Even if this were the case, treatment with a therapy meant to indirectly address this disruption might eventually limit its own efficacy over time. Moreover, to fully unleash the potential of antibodies against misfolded SOD1, it is essential to additionally target and clear misfolded SOD1 within the brain.^349^ This, in turn, necessitates the antibody’s ability to permeate the BBB.

Pozzi et al.^137^ developed a mAb called E6, which targets the RNA recognition motif (RRM1) domain of TDP43. Western blot analysis and immunofluorescence indicated the internalization of the antibody into neuroblastoma mouse cells (Neuro2A). In a cellular model of NF-κB-induced microglial hyperactivation, E6 treatment resulted in approximately 35% reduction in NF-κB in microglia.^137^ Additionally, the antibody reduced cytoplasmic TDP43 in Neuro2A cells, modeling TDP43 pathology, likely through targeting to proteasomes and lysosome-dependent mechanisms.^137^ However, intranasal administration to TDP43A315T tg mice showed location in blood vessels rather than penetrating the CNS parenchyma, as assessed through immunofluorescence imaging techniques,^137^ as mentioned before. In contrast, i.p. treatment resulted in marked detection of the mAb in the third ventricle,^137^ most likely due to diffusion through fenestrated capillaries of the median eminence or via the circumventricular organs surrounding the third ventricle. Considering the significant reductions in nuclear and pathological levels of p65 NF-κB in motor neurons after intrathecal and intracerebroventricular injection of E6 into TDP43A315T tg mice,^137^ there exists the potential for improved efficacy following peripheral injection by enhancing BBB penetration. This enhancement could lead to increased effectiveness when administered less invasively through i.p. or i.v. routes.

Tofersen is an antisense oligonucleotide (ASO) targeting SOD1 mRNA,^368^ leading to the degradation of the heteroduplex mediated by RNase H1.^369^ Intrathecal and intraventricular injection of tofersen in human SOD1 tg rats or mice resulted in a reduction in SOD1 mRNA and misfolded SOD1 levels in the spinal cord, delaying disease onset and extending survival in mouse and rat models.^368^ These promising results prompted the initiation of a phase I/II clinical trial, where ALS patients with SOD1 mutations were treated with tofersen via intrathecal administration.^370^ The study revealed a significant reduction in the SOD1 concentration in the CSF upon tofersen treatment. However, while a correlation between brain and CSF SOD1 concentrations is reported in rats, there is no such correlation in humans.^371^ Subsequent phase III clinical trials also demonstrated CSF SOD1 reduction with intrathecal tofersen treatment, along with a reduction in neurofilament light chain levels in plasma, a biomarker for ALS progression and neurodegeneration.^372–375^ Notably, participants treated with tofersen at trial entry showed higher efficacy than those with delayed treatment, suggesting higher efficacy in early stages of the disease.^372^ Thus, an additional phase III clinical trial has commenced to evaluate the efficacy of tofersen in presymptomatic SOD1 variant carriers, with completion expected in 2027 (NCT04856982).^376^ Although treatment with tofersen did not improve clinical end points, such as slow vital capacity and hand-held dynamometry,^372^ tofersen was recently approved by the FDA for the treatment of ALS patients with SOD1 mutations.^377^ A significant proportion of the adverse effects caused by tofersen are associated with intrathecal administration. Therefore, enhancing the delivery of ASOs into the brain following peripheral administration by improving BBB permeability—for example, through encapsulation in specially designed micelles^378^ or other methods discussed in previous sections—has the potential to markedly enhance both patient well-being and treatment efficacy.

## 6 Discussion and Conclusions

The prevalence of neurodegenerative diseases is increasingly rising, leading to high costs for healthcare systems. Besides their complex and incompletely understood pathologies, the development of therapeutic approaches is hampered by the target′s location in the CNS, a highly protected compartment. The BBB is a tight barrier that strictly shields most brain regions. Biologics, particularly mAbs, have achieved significant success in treating autoimmune diseases and other diseases. Similarly, in neurodegenerative diseases, most novel therapeutics under development belong to this molecular class, and their high affinity and specificity may compensate for the overall low permeability of the brain. A broad variety of *in vitro* and *in vivo* methods have been established to estimate exposure to the drug in the brain, and preclinical *in vitro* studies can deliver a wealth of information. The selection of assay parameters, such as cell types, shear stress, 3D structure, or detection methods, can markedly influence the significance of the findings, leading to a low *in vitro*–*in vivo* correlation. It is therefore highly recommended that different *in vitro* methods be assessed consecutively to verify the BBB permeability before proceeding to *in vivo* experiments.

Many *in vivo* methods are invasive and therefore conducted in animal models, where their complexity might limit their informative value. Measuring the concentration of a substance in the CSF following peripheral administration does not reflect the concentration in the brain due to the CSF–brain parenchyma barrier. Determining the concentration of a macromolecule in the brain requires intracardiac perfusion along with capillary depletion or microdialysis techniques to avoid false-positive results.

Therefore, it is not surprising that no direct BBB penetration is determined for the majority of biologics in development; rather their assessment is focused on clinical outcomes in animal models following peripheral administration,^381–383^ which is an indirect indicator of brain exposure. However, this strategy runs into problems when clinical efficacy is limited. Unfortunately, limited efficacy is the case for most therapeutics developed to treat neurodegenerative diseases; it needs to be ruled out that it is due to a lack of BBB permeability and, therefore, insufficient drug concentration. Another reason for the lack of efficacy might be the late onset of symptoms after substantial degeneration has already occurred. This means that the treatment is initiated too late. To tackle this challenge, research is focused on the identification of biomarkers for early detection. However, the selection of meaningful serum biomarkers (e.g., serum neurofilament light chain or serum aSyn) is also subject to debate, as the correlation between serum levels and brain levels is complex. The process of “reverse transport” of molecules from the brain to the plasma, via the BBB or the brain–CSF interface, is complex and can be influenced by various factors in healthy subjects, and the integrity of the BBB can be altered in neurodegenerative diseases.

As we strive to unlock the full potential of macromolecular therapeutics, three factors have to be tackled in parallel: (1) the development of biomarkers that allow early identification of the neurodegeneration, (2) the development of reliable methods for detection of exposure of the brain to therapeutic compounds, and (3) the development of strategies that can be used by all types of molecular classes of therapeutics to cross the BBB. In this review, we have focused on current methods for tackling BBB permeability by macromolecules, especially those that involve chemical modification of the therapeutic compounds. There are many more approaches for tackling BBB permeability, which we foresee gaining importance, for instance, the use of machine learning methods to predict BBB permeability, for example, for peptides.^384^ This technology will become a major player with an increasing data availability. Other technologies focus on physically and temporarily opening of the BBB, using microbubble-assisted focused ultrasound; however, these methods bear the risk of transient inflammatory responses, flash edema, and intracerebral hemorrhage. Nanomedicine has also been used to permeabilize the BBB to macromolecules.

In conclusion, collaborative efforts in neuroscience, pharmacology, and nanomedicine are essential to overcoming the BBB permeability challenge. This step is pivotal in realizing transformative treatments for neurodegenerative diseases, offering hope for improved patient outcomes in the face of these complex and devastating conditions.

## Abbreviations

11C-PiB: carbon-11-labeled Pittsburgh compound B
2D: 2-dimensional
3D: 3-dimensional
βAPP: gene encoding for amyloid-beta precursor protein
Aβ: β-amyloid
Ab: antibody
ABC: transporter
ATP: binding cassette transporter
AC: astrocyte
AD: Alzheimer’s disease
ADNP: activity-dependent neuroprotective protein
AE: astrocyte end feet
AH: adenohypophysis
ALS: amyotrophic lateral sclerosis
AMT: adsorptive-mediated transcytosis
ApoE4: gene encoding for apolipoprotein E
APP: amyloid-beta precursor protein
ARIA: amyloid-related imaging abnormalities
ASO: antisense oligonucleotide
aSyn: α-synuclein
AUC: area under the curve
BACE-1: β-secretase 1
BBB: blood–brain barrier
Bcl-xL: B-cell lymphoma-extra-large
BEC: brain endothelial cells
CAT1: cationic amino acid transporter 1
CCV: clathrin-coated vesicle
CD14: cluster of differentiation-14
CHT: choline transporter
CMT: carrier-mediated transport
CNS: central nervous system
CNT2: concentrative nucleoside transporter 2
CPP: cell-penetrating peptide
CSF: cerebrospinal fluid
CTX: chlorotoxin
DEN2C: capsid protein of dengue virus type-2
DLS: dorsolateral striatum
EAAT: excitatory amino acid transporter
EE: early endosome
ELISA: enzyme-linked immunosorbent assay
EpC: ependymal cell
ER: endoplasmic reticulum
FC: fenestrated capillary
Fc: fragment crystallizable region (of antibodies)
FcRn: neonatal Fcγ-Receptor
FDA: Food and Drug Administration
GFP: green fluorescent protein
GLUT1: glucose transporter type 1
GP: glycopeptide
gp120: glycoprotein-120
hCMEC/D3: human cerebral microvasculature endothelial cells/D3
HER2: human epidermal growth factor receptor-2
HIV-1: human immunodeficiency virus-1
HTS: high-throughput screening
ICR: Institute of Cancer Research
ID/g: injected dose per gram of brain tissue
IGF1/2R: insulin-like growth factor-1/-2 receptor
IgG: immunoglobulin G
iPSC: induced pluripotent stem cells
LAT1: L-type amino acid transporter 1
LC-MS: liquid chromatography–mass spectrometry
LC-MS/MS: liquid chromatography-tandem mass spectrometry
LDL receptor: low density lipoprotein receptor
LE: late endosome
Lep: leptin
LepR: leptin receptor
LRP-1: low-density lipoprotein receptor-related protein-1
mAb: monoclonal antibody
MAPK: mitogen-activated protein kinases
MCT1: monocarboxylate transporter 1
MDR1: multidrug resistance protein 1
MDS-UPDRS: Movement Disorder Society-sponsored revision of the Unified Parkinson’s Disease Rating Scale
mRNA: messenger ribonucleic acid
MSH: melanocyte-stimulating hormone
MTf: melanotransferrin
MTfp: melanotransferrin peptide
MW: molecular weight
NADPH: nicotinamide adenine dinucleotide phosphate + hydrogen
NF-κB: nuclear factor ‘kappa-light-chain-enhancer’ of activated B-cells
NH: neurohypophysis
NMDA: *N*-methyl-D-aspartate
NPs: nanoparticles
OAT3: organic anion transporter 3
P-gp: P-glycoprotein
PACAP: pituitary adenylate cyclase-activating polypeptide
PAMPA: parallel artificial membrane permeation assay
PC: pericyte
PD: Parkinson's disease
PEG: polyethylene glycol
PET: positron emission tomography
PLGA: poly­(lactide-*co*-glycolic acid)
PS1: presenilin-1
PSEN1/2: gene encoding for presenilin-1 and -2
PT: passive transport
RIA: radioimmunoassay
RGD: arginine-glycine-aspartate
RMT: receptor-mediated transcytosis
RRM1: RNA recognition motif
sandwich ECL: sandwich-type electrochemiluminescence
SE: sorting endosome
SEC: size exclusion chromatography
siRNA: small interfering ribonucleic acid
sMTf: soluble melanotransferrin
SOD1: superoxide dismutase-1
SPECT: single-photon emission computed tomography
Tat: transactivator of transcription
TAUT: taurine transporter
TBI: traumatic brain injury
TC: tanycyte
TDP43: TAR DNA binding protein 43
TEER: transepithelial/-endothelial electrical resistance
TfR: transferrin receptor
tg: transgenic
TJ: tight junction
